# The Evolution of Cell-to-Cell Communication in a Sporulating Bacterium

**DOI:** 10.1371/journal.pcbi.1002818

**Published:** 2012-12-20

**Authors:** Jordi van Gestel, Martin A. Nowak, Corina E. Tarnita

**Affiliations:** 1 Theoretical Biology Group, Centre for Ecological and Evolutionary Studies, University of Groningen, Groningen, The Netherlands; 2 Program for Evolutionary Dynamics, Department of Mathematics, Department of Organismic and Evolutionary Biology, Harvard University, Cambridge, Massachusetts, United States of America; 3 Harvard Society of Fellows, Harvard University, Cambridge, Massachusetts, United States of America; 4 Department of Ecology and Evolutionary Biology, Princeton University, Princeton, New Jersey, United States of America; University of British Columbia, Vancouver, Canada

## Abstract

Traditionally microorganisms were considered to be autonomous organisms that could be studied in isolation. However, over the last decades cell-to-cell communication has been found to be ubiquitous. By secreting molecular signals in the extracellular environment microorganisms can indirectly assess the cell density and respond in accordance. In one of the best-studied microorganisms, *Bacillus subtilis*, the differentiation processes into a number of distinct cell types have been shown to depend on cell-to-cell communication. One of these cell types is the spore. Spores are metabolically inactive cells that are highly resistant against environmental stress. The onset of sporulation is dependent on cell-to-cell communication, as well as on a number of other environmental cues. By using individual-based simulations we examine when cell-to-cell communication that is involved in the onset of sporulation can evolve. We show that it evolves when three basic premises are satisfied. First, the population of cells has to affect the nutrient conditions. Second, there should be a time-lag between the moment that a cell decides to sporulate and the moment that it turns into a mature spore. Third, there has to be environmental variation. Cell-to-cell communication is a strategy to cope with environmental variation, by allowing cells to predict future environmental conditions. As a consequence, cells can anticipate environmental stress by initiating sporulation. Furthermore, signal production could be considered a cooperative trait and therefore evolves when it is not too costly to produce signal and when there are recurrent colony bottlenecks, which facilitate assortment. Finally, we also show that cell-to-cell communication can drive ecological diversification. Different ecotypes can evolve and be maintained due to frequency-dependent selection.

## Author Summary

Biological systems are characterized by communication; humans talk, insects produce pheromones and birds sing. Over the last decades it has been shown that even the simplest organisms on earth, the bacteria, communicate. Despite the prevalence of communication, it is often hard to explain how communicative systems evolve. In bacteria, communication results from the secretion of molecular signals that accumulate in the environment. Cells can assess the concentration of these signals, which indicate cell density, and respond in accordance. This form of cell-to-cell communication is responsible for the regulation of numerous bacterial behaviors, such as sporulation. Spores are metabolically inactive cells that are highly resistant against environmental stress. It is adaptive for a cell to sporulate when it struggles to survive. We show, via individual-based simulations, that cell-to-cell communication evolves because it allows cells to predict future environmental conditions. As a consequence, cells are capable of anticipating environmental stress by initiating sporulation before conditions are actually harmful. Furthermore, our model shows that cell-to-cell communication can even drive ecological diversification, since it facilitates the evolution of individuals that specialize on distinct ecological conditions.

## Introduction

Complex systems in biology often come about through the communication of their parts, such as pheromone communication in insect societies and language in humans. Communication has been found to be ubiquitous in microorganisms as well [1]–[4]. Due to self-produced molecular signals that are secreted in the environment, cells can monitor the population density, which can quantitatively affect a cell's gene expression or trigger a differentiation process. In 1994, Fuqua and colleagues were the first to characterize this form of cell-to-cell communication as quorum-sensing signaling [5]. Quorum-sensing signaling has been shown to regulate a multitude of bacterial processes, such as extracellular enzyme production, antibiotic production and biofilm formation [6]–[11]. In one of the best-studied microorganisms, *Bacillus subtilis*, the differentiation of a number of cell types has been shown to depend on cell-to-cell communication [12]–[14]. These cell types emerge during the developmental process of biofilm formation and are presumably needed to survive the harsh environmental conditions that are present in the soil [10], [15], [16]. The most remarkable survival strategy among these cell types is that of the spore [17], [18].

A spore is a metabolically inactive cell that compartmentalized its DNA together with some essential proteins to survive starvation or other environmental stressors [18], [19]. Spore formation is an energy-expensive process that can take 6 to 8 hours and involves the expression of hundreds of genes [19], [20]. The initiation of sporulation is primarily dependent on the activation of a single transcription factor called Spo0A [14], [21]–[24]. When the level of activated Spo0A is sufficiently high, the sporulation process will be initiated [25]–[28]. The level of activated Spo0A is indirectly affected by a number of environmental and physiological cues, of which some are self-produced quorum-sensing signals [13], [22], [29]. These signals are assumed to accumulate in the environment and thereby give an indication of the cell density. As a consequence, the fraction of cells that initiate sporulation is higher for higher cell densities [29]–[33]. Even though these quorum-sensing signals affect the proportion of cells that initiate sporulation, they themselves are not sufficient for initiating sporulation since starvation is absolutely required [34]–[36]. Bischofs and colleagues (2009) mathematically modeled the regulatory mechanisms that integrate the quorum-sensing signals with other environmental cues, including those that are indicative of starvation [36]. They showed that the quorum-sensing signals allow for a density-dependent normalization of certain environmental cues. For example, when a cell can sense the amount of nutrients that are left in the environment, quorum-sensing signaling makes it possible to estimate the amount of nutrients that are left per cell. They concluded that these density-dependent normalizations might be adaptive for cellular decision-making, such as determining when to initiate sporulation (see also [34]).

However, despite the detailed knowledge of the regulatory mechanisms that underlie the sporulation process, little is known about their evolutionary origin. Why does cell-to-cell communication evolve and under which ecological and developmental conditions is it selected for? Here we examine, by using individual-based simulations, how three conditions, which inevitably relate to sporulation [15], [19], [20], [34], [37], [38], affect the evolution of cell-to-cell communication: environmental variation in nutrient conditions, costs of sporulation and time expenditure of sporulation. Even though our model is inspired by sporulation in *B. subtilis*, it is aimed to be conceptual and therefore does not include mechanistic details. The model is made such that it allows for the evolution of various developmental strategies, in which a cell's sensitivity and response to environmental cues can evolve.

Throughout the paper we discuss different versions of our model, which gradually increase in complexity. First we study the evolution of cell-to-cell communication under clonally-growing colonies. Next we allow for within colony-variation by initiating colonies with multiple individuals. Under these conditions multiple ecotypes evolve that transiently coexist over time due to negative frequency-dependent selection. Finally, we examine the evolution of cell-to-cell communication when signal production is costly. Under these conditions cooperative dilemmas emerge naturally and we find that different ecotypes evolve, which use different communicative strategies to time the onset of sporulation. The evolutionary significance of these strategies can only be understood by considering their ecological context.

## Model

We assume that cells are scattered throughout the soil. Only in a few locations these cells can grow and form colonies, because only in these areas there are nutrients available to do so. During colony growth cells consume nutrients in order to perform cell division and cell differentiation. A cell can differentiate into two cell types—a signal-producing cell or a spore—or it could remain undifferentiated. Eventually, all the nutrients will be depleted and a colony enters a starvation period. This period can only be survived by the spores. It is therefore crucial for a cell to initiate sporulation on time (i.e. when the nutrients that are needed to complete the sporulation process are still available). To decide when to initiate sporulation a cell could make use of two environmental cues: the nutrient concentration and the amount of quorum-sensing signal. The spores that eventually survive the starvation period migrate and germinate in new nutrient rich areas, where they form new colonies. Over evolutionary time, a cell's responsiveness to the environmental cues can evolve and thereby the timing of sporulation can evolve as well. We examine under which ecological and developmental conditions there is selection for cells that use quorum-sensing signaling to time the onset of sporulation. The system is studied by using individual-based simulations, which we describe in the following paragraphs.

**Figure 1:**
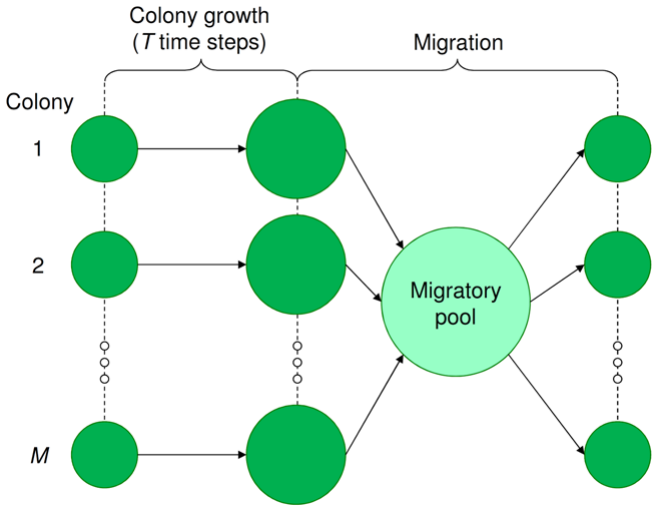
Nutritional cycle and population structure. The colonies (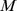 in total) are first allowed to grow for a fixed number of time steps (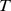). Then, all individuals (spores and cells) enter migration, forming a single migratory pool. From this pool, 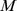 new colonies are established by taking, for each colony separately, 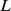 random individuals. The complete cycle from the establishment of the colonies to the eventual migration of the individuals is called the nutritional cycle and is repeated over time. Notice that for clonally growing colonies 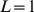, hence only a single genotype establishes a new colony.

We assume that the population of cells is divided into 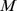 subpopulations, each representing a colony (i.e. biofilm or pellicle). Each colony is established by 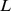 individuals. A colony is said to grow clonally when it is established by only one individual (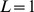). At the onset of colony growth there is a single nutrient input, which for each colony is taken from a normal distribution that is given by 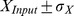. Thus, the nutrient could be different for each colony. After receiving the nutrient input colonies are allowed to grow for a fixed number of time steps (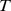); during this period cells consume nutrients in order to perform cell division and differentiation. At the end of a nutritional cycle all individuals (cells and spores) enter migration. The nutritional cycles of all colonies are synchronized such that the individuals from all colonies enter migration at the same time, forming a single migratory pool (see figure 1). Since migration occurs passively, we assume that all individuals have the same chance to establish a new colony. Thus, 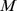 new colonies are established by choosing, for each colony separately, 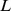 random individuals from the migratory pool. After this, the new colonies simultaneously start the next nutritional cycle.

Within a nutritional cycle three different cellular processes can occur at any time step (for each cell in the colony). First, a cell gets the opportunity to differentiate. A cell can differentiate into two different cell types—a signal-producing cell or a spore—or it could remain undifferentiated. A signal-producing cell secretes a fixed amount of signal in the environment. The more cells that produce signal, the higher the amount of extracellular signal. At the same time, the signal is degraded with a fixed rate 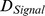. Thus, the amount of signal changes over time depending on the number of cells that are producing it. A cell could also initiate sporulation. Sporulation is an irreversible process that takes a fixed number of time steps (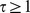) and during which a fixed amount of nutrients is consumed (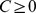), which is needed for making the spore. Thus, a sporulating cell consumes 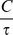 nutrients per time step. When there is an insufficient amount of nutrients in the environment, the sporulation process cannot be completed; in this case a cell inevitably dies. After completing the sporulation process, a mature and resistant spore is formed. A spore cannot divide, but has a much lower death rate than a cell. A spore germinates at the onset of a new nutritional cycle. Since sporulation requires 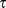 time steps, a cell can be in one, out of 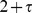, phenotypic states. It can be an undifferentiated cell, a signal producing cell or a sporulating cell, of which the latter is subsequently composed of 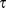 states that indicate the number of time steps a cell has been sporulating (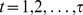). At the final time step of sporulation (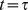) a cell turns into a spore. The cell's decision to differentiate into a signal-producing cell or spore depends in our model on two environmental cues—the amount of nutrients and signal—and on a cell's genotype (which we describe later).

The second cellular process that a cell can undergo, after having had the opportunity to differentiate, is division. All cells, excluding spores, have a certain chance of dividing. This chance is dependent on the amount of nutrients that are present in the environment (for details see equation S1). The more nutrients that are present in the environment, the greater the chance of cell division, with a maximum chance of 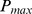. During each cell division a fixed amount of nutrients (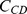) is consumed. At each cell division there is a certain probability that the dividing cell incurs a mutation (the mutation process is described later).

The third and last cellular process that can occur at any particular time step is that of cell death. Both cells and spores have a fixed chance of dying, which is independent of the nutrient concentration. The death rate of a spore is much lower than that of a cell (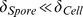). Hence, it is better to be a cell when nutrients are plentiful, because the chance of having cell division outweighs the chance of having cell death. On the contrary, when the nutrients are depleted, it is better to be a spore because spores have a smaller chance of dying than cells. The fitness of a genotype therefore depends on the timing of sporulation. When a genotype sporulates too early—at a nutrient concentration that is too high—it loses reproductive potential, since not all the nutrients are utilized. When a genotype sporulates too late—at a nutrient concentration that is too low—it has an increased risk of dying, especially when, due to nutrient scarcity, the sporulation process cannot be completed.

**Figure 2:**
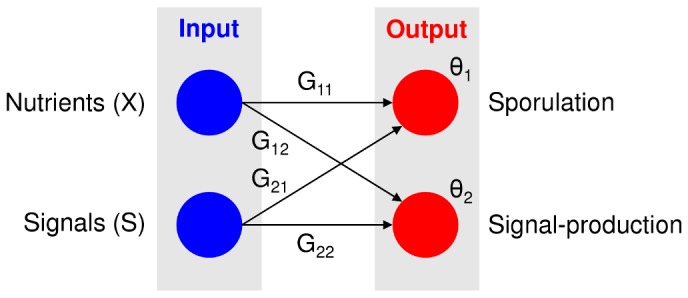
Regulatory network that regulates cell differentiation. The left side shows the environmental cues that a cell can sense (the *blue* nodes): the nutrient concentration (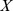) and the amount of signal (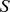). The right side shows the different cell types into which a cell can differentiate (the *red* nodes). A cell could differentiate into a sporulating or signal-producing cell. Each connection (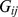) shows how the associated environmental cue affects the differentiation process (*black* arrows). Each activation threshold (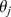) shows how much stimulus is required before the associated cell differentiation process occurs. The regulatory network corresponds to inequality 1a and 1b, which are described in the main text.

A crucial part of the model is the cell differentiation process. We aim to model it such that various developmental strategies can evolve. This requires to have sufficient degrees of freedom. On the other hand, we want to restrict the number of evolvable variables, in order to keep the model simple and tractable. The combination of these requirements resulted in a cell differentiation process that could be described by two Boolean decision-making steps, which are affected by the amount of nutrients and signal. The cell should decide to initiate sporulation or not and when it does not sporulate, a cell should decide if it wants to produce signal or not. These two decisions can be expressed by the following two inequalities (see figure 2):
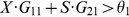
(1a)

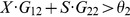
(1b)Inequality 1a shows when a cell initiates sporulation and inequality 1b shows when a cell initiates signal production. We assume that the decision to initiate sporulation is dominant over the decision to produce signal. Thus when both inequalities hold, only the sporulation process is initiated. The left hand side of each inequality contains the environmental cues: the amount of nutrients (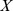) and the amount of extracellular signal (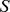). Since nutrients are consumed and signal can be produced and degraded over time, the values of these environmental cues change during colony growth. The effect of an environmental cue on the differentiation process depends on what we call the connection weight, 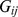; here 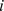 is the environmental cue (1 is the amount of nutrients and 2 is the amount of signal in the environment) that is affecting differentiation process 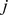 (1 is sporulation and 2 is signal production). For example, 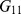 determines how the amount of nutrients affects the initiation of sporulation. When a connection weight is positive, its corresponding environmental cue stimulates the differentiation process. When the connection weight is negative, the environmental cue inhibits the differentiation process. The absolute value of a connection weight shows the impact that a certain environmental cue has on the differentiation process. The right hand side of both inequalities is the activation threshold, 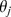; here 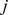 is the differentiation process to which the activation threshold belongs (1 is sporulation and 2 is signal production). The activation threshold shows how much stimulus from the environmental cues is required before the differentiation process is initiated. For example, when 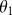 is positive a cell only sporulates when the stimulus from the nutrients (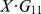) plus the stimulus from the signal (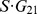) is bigger than the activation threshold (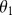). On the contrary, when 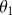 is negative a cell sporulates by default (when 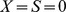) and sporulation can only be prevented if the environmental cues inhibit the sporulation process (i.e. negative connection weights). The activation thresholds could be viewed as a normalization of the connection weights. Namely, one could divide both sides of inequality 1a and 1b by the absolute values of, respectively, 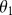 and 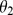, without altering the behavior of a genotype. Therefore the model could be simplified by fixing the activation thresholds (i.e. preventing mutations to occur in the activation thresholds), as long as it does not affect the strategies that can evolve. In the first two sections of the results we applied this simplification to the model and only allowed the connection weights to mutate. To show that this simplification did not affect the evolutionary outcome of the model we performed all simulations under non-simplified conditions and show the results in the supplementary information (figure S3). In the last section we did not fix the activation thresholds, because when signal production is assumed to be costly, the evolutionary outcome would be constrained by fixing the activation thresholds. We call the collection of connection weights (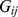) and activation thresholds (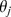) the genotype of an individual. In essence, the genotype describes how a cell responds to each combination of environmental cues.

When a cell division occurs each of the genotypic variables (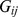 and 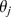) has a certain chance to mutate (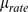). When a mutation occurs, a small value taken from the normal distribution 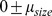 is added to the genotypic variable. Every mutation is taken independently from the same normal distribution, irrespective of the genotypic variable that mutates. All evolutionary simulations are initiated with the same monomorphic population of cells that do not produce signal and are not sensitive to it (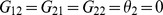). In addition, the initial cells are assumed to sporulate, to prevent the population from going extinct. The initial cells sporulate at a nutrient concentration of 500 (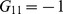 and 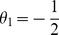; all input variables that are perceived by the cells are divided by 1000 as normalization, which is done consistently throughout the paper). Similar results would however be obtained if sporulation would occur at another nutrient concentration, as long as the initial population does not go extinct in the first growth cycle. By assuming that both 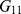 and 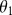 are negative, we assume that nutrients inhibit the sporulation process and that when this inhibition is too weak (e.g. when 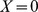) a cell initiates sporulation. Thus, we are not examining the evolution of sporulation, but the evolution of cell-to-cell communication as a mechanism to time the onset of sporulation.

## Results

A cell should turn into a spore when the growth rate of a spore exceeds that of a cell. The effective growth rate is given by the birth rate (i.e. chance of cell division; equation S1) minus the death rate (i.e. chance of cell death; 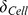 and 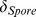 for respectively cells and spores). Since a spore cannot divide, its effective growth rate is 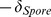, which is approximately equal to 0 (assuming that 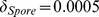). A cell should therefore turn into a spore when the chance of having cell death exceeds the chance of having cell division. The chance of cell division is subsequently dependent on the nutrient concentration (see equation S1). Thus, there is a critical nutrient concentration at which a cell should turn into a spore (see equation S2). However, sporulation costs time and during sporulation nutrients are consumed [19], [20]. In other words, the decision to sporulate has to be made in advance, before the critical nutrient concentration is reached. We examine why and when a cell uses quorum-sensing signals for its decision to sporulate. Moreover, we examine under which conditions cell-to-cell communication evolves. This is done for different variants of the model with increasing complexity. First, we examine if cell-to-cell communication evolves under the assumption that colonies grow clonally. Second, we examine how within-colony variation affects the evolution of cell-to-cell communication. Third and last, we examine if cell-to-cell communication evolves when signal production is costly.

### Clonally growing colonies

**Figure 3:**
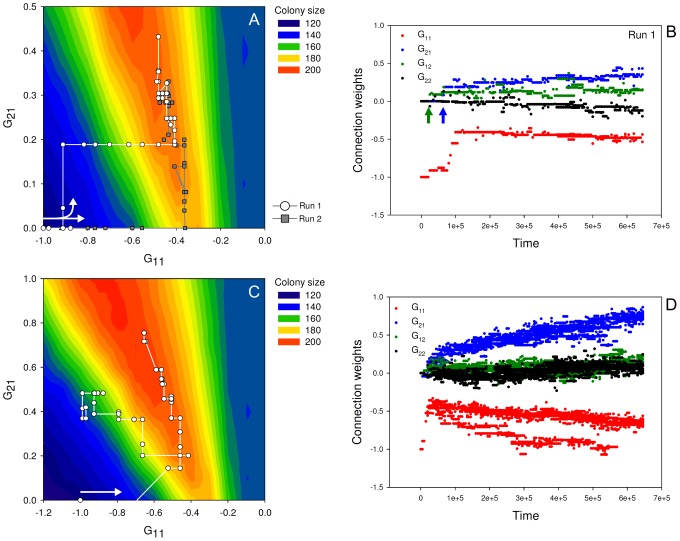
The evolution of cell-to-cell communication under clonal and non-clonal growth conditions. The upper panel (plot A and B) shows the clonal growth conditions (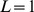). The lower panel (plot C and D) shows the non-clonal growth conditions (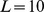). The left plot in each panel (plot A & C) shows the evolutionary trajectory (645.000 time steps with 5.000 time step intervals) plotted on an adaptive landscape. Thereby illustrating the evolution of cell-to-cell communication. The right plot (plot B & D) in each panel shows the connection weights of the most-abundant genotypes (present in the population in more than 100 copies). These figures thereby show both the evolution of signal sensitivity and signal production. In addition, they show how sporulation depends on the nutrient concentration (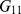). The adaptive landscapes (background coloration of plot A and C) are generated by growing each genotype—meaning each combination of 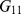 and 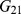
—clonally and taking the average colony size at the end of a nutritional cycle as fitness measurement (assuming that 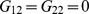, 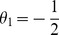 and 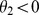). The *white* arrows within plot A and C show the onset of the evolutionary trajectory, as well as the direction of evolution. The arrows in plot B indicate when signal production (*green* arrow) and signal sensitivity (*blue* arrow) evolved. The parameter settings are the following: 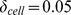, 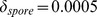, 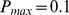, 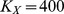, 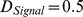, 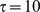, 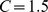, 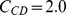, 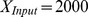, 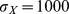, 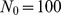, 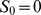, 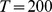, 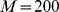, 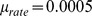 and 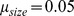.

In this section we examine the evolution of cell-to-cell communication under the assumption that colonies grow clonally, meaning that colonies are initiated by a single individual (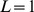). Genetic variation can only arise in these colonies via mutations. Moreover, for simplicity as explained before, we also assume that only the connection weights (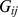) can mutate (similar results are however obtained when the activation thresholds are allowed to mutate as well; see figure S3). Under these conditions, the timing of sporulation depends on 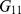 and 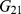 and the differentiation into a signal-producing cell solely depends on 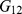 and 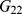 (the activation thresholds, 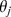, are fixed over evolutionary time). To evolve cell-to-cell communication a cell should acquire two properties over evolutionary time. First, a cell should produce signal. Thus, before initiating sporulation a cell has to differentiate into a signal-producing cell. Second, a cell should be sensitive to the signal (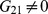), meaning that the nutrient concentration at which a cell initiates sporulation has to depend on the amount of signal. Irrespectively of the order in which these properties evolve, when both are present there is cell-to-cell communication. To examine if both properties can evolve in our model, we ran individual-based simulations that were initiated with a monomorphic population of cells that did not produce signal and were not sensitive to the signal (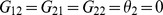). Figure 3A shows two independent evolutionary trajectories projected on an adaptive landscape (for more replicates see figure S1).

The adaptive landscape is constructed by showing for each possible genotype—meaning each combination of 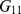 and 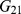
—the average colony size that is obtained at the end of a nutritional cycle. When solely examining the adaptive landscape, one expects that cell-to-cell communication would evolve, because the best-performing genotypes that are signal-sensitive (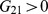) have a higher fitness than those that are signal-insensitive (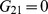). The two evolutionary trajectories that are plotted on the adaptive landscape are called run 1 and run 2 (both runs were performed under the same parameter settings). In both runs cell-to-cell communication evolved, which means that both signal-production and signal-sensitivity evolved. The evolutionary trajectories of figure 3A and S1 closely match the adaptive landscape and hence the adaptive landscape can be used to predict the outcome of evolution. The adaptive landscape only shows the selective advantage of cell-to-cell communication for 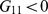 and 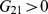 since nothing interesting happens outside this quadrant. In other words, nutrients are expected to inhibit sporulation (i.e. a cell only sporulates when there is nutrient scarcity), while signal is expected to stimulate sporulation (i.e. a cell sporulates earlier when it occurs in a bigger population). A limitation of the adaptive landscape of figure 3A is that it does not show the other two connection weights, 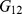 and 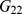. 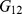 and 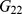 determine when a cell differentiates into a signal-producing cell (see figure 2). Signal production is, next to signal-sensitivity, essential for the evolution of cell-to-cell communication. To examine how signal production evolved we plotted the values of all connection weights (corresponding to the most-abundant genotypes), of run 1, along a time-axis (see figure 3B).

Figure 3B shows that signal production evolves after about 20.000 time steps (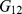 becomes positive; as indicated by the *green arrow*). About 40.000 time steps later signal-sensitivity evolves as well (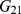 becomes positive; as indicated by the *blue arrow*). In other words, signal production emerges before the occurrence of signal-sensitivity. Hence there was no selective advantage for signal production at the moment it evolved. Signal production evolved because a neutral mutation in 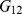 hitchhiked along with a beneficial mutation in 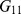. Genetic hitchhiking is relatively prevalent, because there is no genetic recombination. In addition, there are no costs for signal production in this version of the model. Thus, cell-to-cell communication evolves by the sequential evolution of signal production and signal-sensitivity.

**Figure 4:**
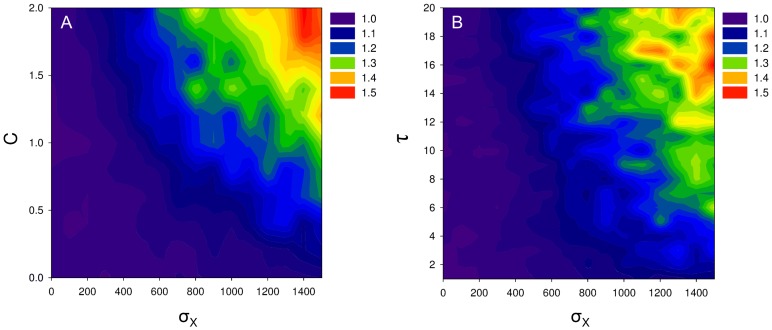
Selective advantage of cell-to-cell communication. Plot A shows the relative fitness benefit of cell-to-cell communication under different parameter conditions of 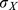 and 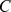 (meaning the amount of environmental variation in the nutrient input and the amount of nutrients required for completing a single sporulation process). Plot B shows the relative fitness benefit of cell-to-cell communication under different parameter conditions of 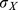 and 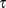 (meaning the amount of environmental variation in the nutrient input and the time-lag between the decision to sporulate and actually being a spore). The relative fitness is defined as the relative colony size of colonies that contain communicative cells over those that do not. Thus, when the relative fitness is bigger than one there is selection for cell-to-cell communication. For plot A we assume that 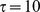 and for plot B we assume that 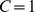, hence the horizontal lines at which 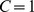 in plot A and 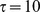 in plot B are replicates of the same parameter conditions. The other parameter settings are the following: 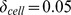, 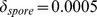, 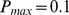, 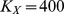, 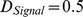, 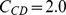, 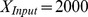, 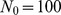, 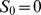 and 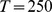.

The question we are interested in though, is why cell-to-cell communication evolved at all. By sensing signal a cell can assess the colony size at the onset of sporulation. This estimate gives an indication of the amount of nutrients that will be consumed by the colony during sporulation. As explained before, a cell should turn into a spore when the chance of having cell death exceeds that of cell division, which is associated with a critical nutrient concentration (for details see equation S2). Since sporulation requires time, a cell has to anticipate or predict if the nutrient concentration at the end of sporulation matches this critical nutrient concentration. To make this prediction it is necessary to assess the amount of nutrients that will be consumed during sporulation. Since the total amount of nutrient consumption depends on the number of cells within a colony, it is advantageous for a cell to sense quorum-sensing signals. When the colony is big, a high amount of nutrients will be consumed during sporulation due to which a cell should initiate sporulation relatively early (i.e. at a high nutrient concentration). On the contrary, when the colony is small, a small amount of nutrients will be consumed and therefore a cell should initiate sporulation relatively late (i.e. at a low nutrient concentration). Thus, cell-to-cell communication allows a cell to predict the total amount of nutrient consumption during sporulation and, thereby, a cell can anticipate future environmental changes. There are three requirements that should be satisfied for cell-to-cell communication to evolve (corresponding to the parameter values in our model; see figure 4): (i) the colony size should affect the nutrient concentration during sporulation by, for example, nutrient consumption (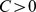); (ii) there should be a time-lag between the moment that a cell decides to sporulate and the moment that it turns into a mature spore (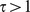); and (iii) there should be environmental variation (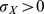). High values of 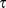, 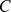 and 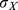 (e.g. 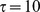, 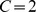 and 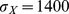) can result in a 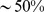 fitness advantage for cells that sense quorum-sensing signals over those that do not (figure 4).

The first requirement for the evolution of cell-to-cell communication is that the colony size should affect the nutrient concentration (figure 4A). For example, when each cell consumes a fixed amount of nutrients during sporulation (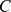), the total nutrient consumption depends on the colony size. When there is no nutrient consumption during sporulation (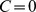) the optimal time at which to initiate sporulation does not depend on the colony size and hence cell-to-cell communication does not evolve. Second, cell-to-cell communication only evolves when there is a time-lag between the moment that a cell decides to sporulate and the moment that it turns into a spore (figure 4B). In other words, sporulation should require time. When sporulation does not require time, there is no need to assess the nutrient consumption since a cell could turn into a spore instantaneously. Thus, cell-to-cell communication only evolves when 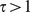. The third and last requirement for the evolution of cell-to-cell communication is the presence of environmental variation (figure 4). When there is no variation (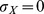), the amount of nutrients at the onset of a nutrient cycle is always the same. As a consequence, the changes in the nutrient concentration over time correlate with those of the colony size, since all colonies are initiated with the same number of cells, which reproduce at the same rate. Under these conditions, the nutrient concentration could be used as an accurate indication of the colony size, which makes the use of quorum-sensing signals superfluous, since these give an indication of the colony size as well. Only when the correlation between the nutrient concentration and colony size is relatively weak, the amount of signal could be used as a unique indication of the colony size. For this reason, there is stronger selection for cell-to-cell communication for higher levels of 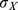. Alternative conditions that weaken the correlation between the colony size and nutrient concentration can have a similar effect. For example, one could vary the initial colony sizes; colonies would still be clonal but different colonies would be initiated by different numbers of cells (see figure S8).

### Within-colony variation

In most laboratory experiments sporulation is studied in isogenic populations. However, it is plausible that multiple genotypes can co-occur in a single colony [39]. In this section we examine how the developmental mechanisms that determine the onset of sporulation evolve when multiple genotypes can initiate a single colony (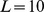). This is done for the same conditions as those described in the previous section (i.e. only the connection weights, 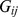, are allowed to mutate; see figure S3 for simulations in which also the activation thresholds could mutate).

In figure 3C the evolutionary trajectory of a single run is shown on the adaptive landscape. Figure 3D shows, for the same evolutionary run, the connection weights of the most-abundant genotypes along a time-axis (for more replicates see figure S2). In contrast to the previous section, there is a bifurcation event during the evolutionary process that results in two coexisting ecotypes (an ecotype is a cluster of genotypes that is adapted to specific ecological condition). One of these ecotypes eventually goes extinct (see figure 3D and S2). Both ecotypes produce quorum-sensing signal and are sensitive to it. The ecotypes only differ in their responsiveness towards the nutritional conditions in the environment (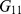). In one ecotype the value of 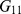 is lower than in the other, meaning that the nutrients more strongly inhibit the sporulation process (see figure 3D and S2). This ecotype is therefore called the late sporulating ecotype (i.e. sporulation is initiated at a low nutrient concentration), while the other one is called the early sporulating ecotype (i.e. sporulation is initiated at a high nutrient concentration).

How can the late and early sporulating ecotypes stably coexist? In the absence of cell-to-cell communication, a genotype can only efficiently make use of the available nutrients for a limited range of nutrient inputs (i.e. nutrient concentration at the onset of a nutritional cycle; see figure S4 and S5B). When the nutrient input is higher than this particular range, a genotype would sporulate too late and when it is lower than this range a genotype would sporulate too early (see figure S4). When a genotype sporulates too early, not all the nutrients will be consumed. The leftovers can be used by other genotypes that sporulate slightly later and co-occur in the same colony. The late sporulating genotypes, in turn, cannot efficiently make use of the nutrients at high nutrient inputs, because they initiate sporulation too late. As a consequence, there is frequency-dependent selection in which the late sporulating ecotype has a selective advantage when the early sporulating ecotype is abundant and *vice versa* (see figure S6). Figure 3D shows that the early sporulating ecotype evolves first and later is accompanied by the late sporulating ecotype.

Over evolutionary time both the early and late sporulating ecotypes become more sensitive to the quorum-sensing signal (increase in 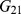) and thereby evolve cell-to-cell communication (figure 3D). In other words, both ecotypes evolve the ability to adjust the timing of sporulation to the nutrient input. This increases the range of nutrient inputs at which an ecotype could efficiently make use of the nutrients (see figure S5C). As a consequence, there is an increasing overlap in the range of nutrient inputs at which both ecotypes grow efficiently, hence strengthening the competition between them. Ultimately, only a single ecotype survives (see figure 3D and S2). This ecotype is a generalist, since it grows efficiently at most nutrient inputs due to the evolved cell-to-cell communication. Thus, over evolutionary time, the evolved specialists—the early and late sporulating ecotypes—are replaced by a generalist—a signaling ecotype—that can grow efficiently at most nutrient inputs.

Not surprisingly, when there is no environmental variation (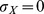), a bifurcation event cannot occur. In that case only a single ecotype evolves that outcompetes all others (see figure S7). Branching is most likely to occur for high levels of 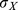 (see figure S7); the same conditions that select for cell-to-cell communication (see figure 3 and 4). Another condition under which a bifurcation event cannot occur is clonal growth, since it hampers the presence of within-colony variation. Within-colony variation allows for competition at the cellular-level and hence for the coexistence of multiple ecotypes. However, allowing for within-colony variation can also result in a conflict between the genotypes that are selected for at the colony-level and those that are selected for at the cellular-level. In particular, when signal production is costly conflicts are expected, since cells that do not produce the costly signal have a fitness advantage at the cellular-level but undermine the performance of the colony. In the next section we examine whether cell-to-cell communication evolves when signal production is costly.

### Costs for signal production

In this section we examine whether cell-to-cell communication can still evolve when signal production is costly. We assume that a signal-producing cell has a reduced chance of dividing by subtracting a fixed value (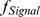) from the chance of having cell division (see equation S3). In contrast to the previous sections, all genotypic variables can mutate, to allow for a wider variety of communicative strategies. In this section we focus on a single representative evolutionary run (for more replicates see figure S9).

**Figure 5:**
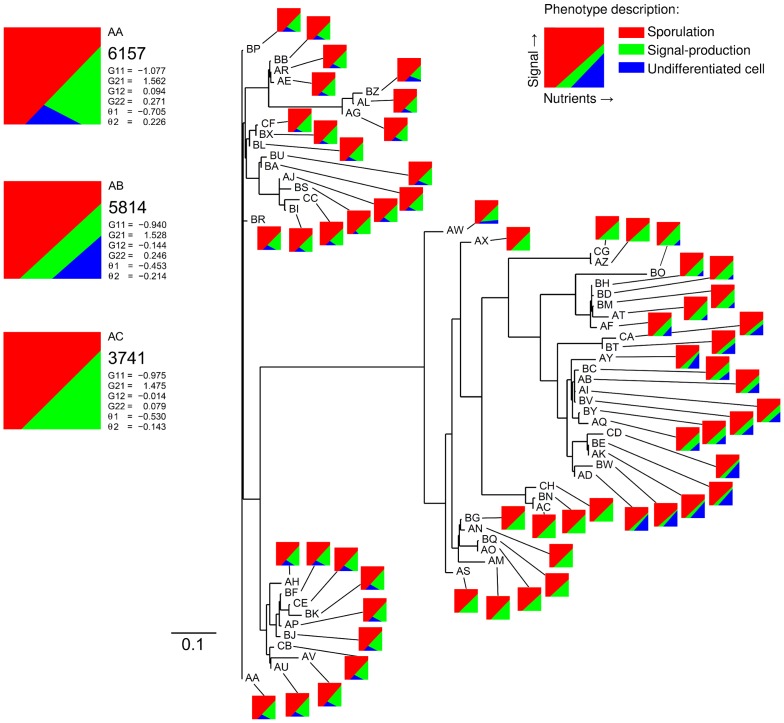
Unrooted phenogram based on the most-abundant genotypes at time step 550.000. This diagram shows the phenotypic population structure at time step 550.000 based on genotypic relatedness. The horizontal lines represent the distances between genotypes. The distance between two genotypes is given by the sum of absolute differences between the connection weights and activation thresholds of both genotypes. Thus, closely related genotypes cluster together in the tree diagram. Only horizontal distances are informative, thus the upper and lower clusters are closer related to each other than either of them are to the biggest cluster of genotypes in the middle. From the tree one cannot infer evolutionary descendance, because it is unrooted. The tree is constructed from the distance matrix of the 60 most-abundant genotypes using the Fitch-Margoliash method (from the PHYLIP v3.69 package). The letter-code that is given to each genotype represents abundance, with ‘AA’ being the most-abundant genotype and ‘CH’ the least-abundant genotype. The three most-abundant genotypes and their associated phenotypes are shown in the upper left corner (AA, AB & AC). For each of these genotypes, we show the abundance, connection weights, activation thresholds and phenotype description. The phenotypes are described by a small diagram that shows the behavior of a cell for different environmental conditions: *red* area is sporulation; *green* area is signal production; and *blue* area is no differentiation. The parameter settings that are used for this simulation are the following: 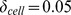, 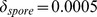, 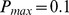, 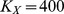, 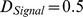, 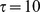, 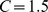, 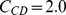, 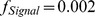, 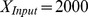, 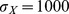, 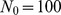, 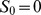, 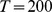, 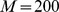, 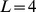, 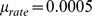 and 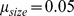.

**Figure 6:**
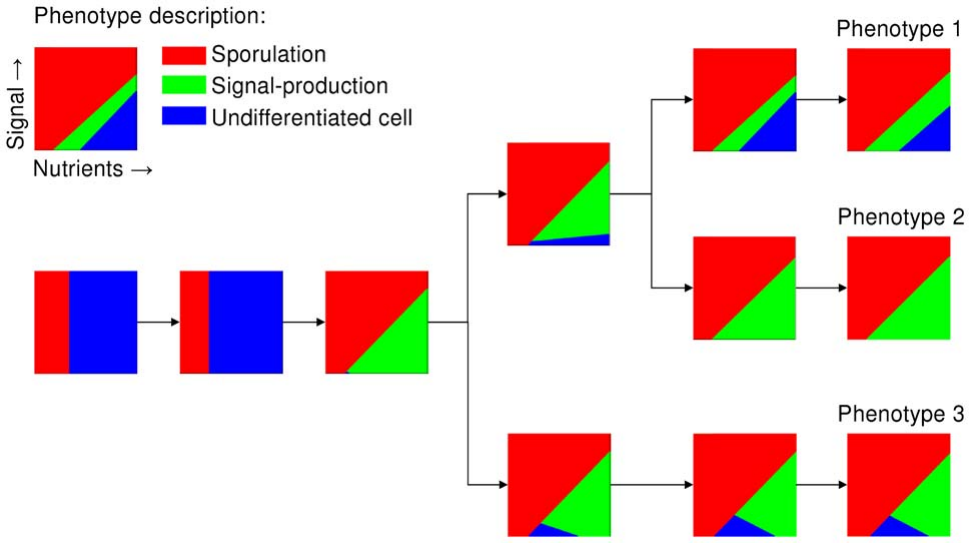
The evolution of different phenotypes shown by an evolutionary tree. The phenotypes that are associated with the three most-abundant genotypes that were present at the end of the simulation (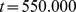) are called phenotype 1, 2 and 3, each belonging to a distinct ecotype. The phenotypes that are projected on the evolutionary tree correspond to the ancestral and evolved genotypes at respectively time step 0, 100.000, 300.000, 400.000, 500.000 and 550.000 (from the left to the right). Each phenotype is shown by a small graph that shows the behavior of a cell for different environmental conditions: *red* area is sporulation; *green* area is signal production; and *blue* area is no differentiation. For the parameter settings see figure 5.

Figure 5 shows the outcome of this evolutionary run, by using a phenogram. The phenogram shows the dissimilarity between genotypes in a population that evolved for 550.000 time steps. The genotypes are named by letter-codes, which are ranked in alphabetic order and represent abundance, with genotype ‘AA’ being the most abundant and genotype ‘CH’ the least. Besides the letter-code, every genotype is connected to a small graph, which shows its phenotype for a range of environmental conditions. The population consists of multiple communicative strategies that cluster together. The three most-abundant genotypes partly reflect these clusters and are shown on the left side of the phenogram. Since, the phenogram does not show evolutionary descendance, the evolutionary lineages of the three most-abundant genotypes were used to construct an evolutionary tree. This tree is shown in figure 6. Hereafter, the phenotypes of the three most-abundant genotypes are called phenotype 1, 2 and 3; corresponding to the order in which they appear in figure 6.

All three phenotypes produce quorum-sensing signal for a range of parameter conditions (shown by the *green* areas in figure 6). Phenotype 2 produces quorum-sensing signal for all environmental conditions, except for those at which it sporulates. Since signal production is costly this phenotype is exploited by phenotype 1 and 3, which lack signal production for respectively high and low nutrient concentrations. As a consequence, phenotype 2 is always selected against at the cellular-level, irrespective of the population composition at the onset of a nutritional cycle. However, phenotype 2 is maintained in the population due to selection at the colony-level, in which the colonies that contain phenotype 2 often have a selective advantage over those that do not contain phenotype 2 (for details see table S1). This selective advantage results from the improved timing of sporulation. Thus, the selection pressures at the colony-level outweigh those at the individual-level. Since the other two phenotypes exploit phenotype 2 for different environmental conditions, they occupy different niches.

**Figure 7:**
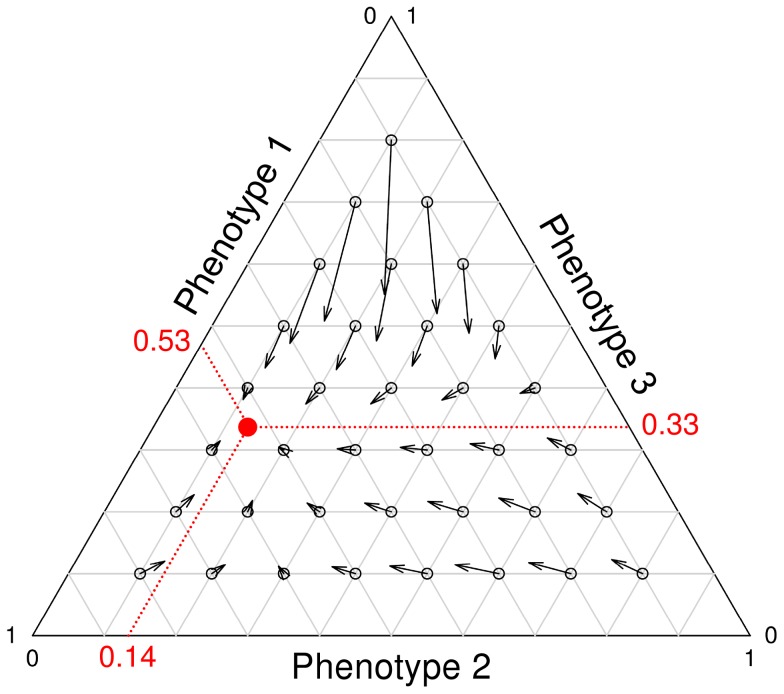
Selection pressures that act on the three most abundant phenotypes. The direction of an arrow shows how the phenotype frequencies change over time. The length of an arrow indicates the speed of this change and hence the strength of selection. The *red* dot shows to the phenotype frequencies at equilibrium (i.e. the population state in which all phenotypes have exactly the same fitness). The frequency changes are determined from the onset of the current nutritional cycle to that of the next nutritional cycle. The calculations therefore include both cellular-level and colony-level selection. For the parameter settings see figure 5.

Figure 7 shows the selection pressures that act on each phenotype, given the frequency at which each phenotype occurs in the population (frequency over all colonies). The fitness measurements include the selection processes at the cellular- and colony-level. All phenotypes have a selective advantage when they are present in a low overall frequency. Thus, negative frequency-dependent selection is responsible for the stable coexistence of the three phenotypes. Since the three phenotypes are subject to a continuing process of evolution, it is unlikely that these specific phenotypes would coexist forever. Frequency-dependent selection does however assure the coexistence of multiple ecotypes, as shown by figure 5 and S9.

**Figure 8:**
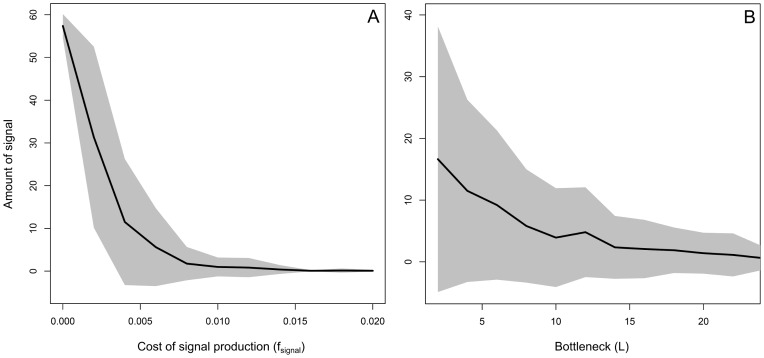
Evolution of signal production under various levels of signal costs and colony bottleneck sizes. The plots show the amount of signal that is present in a population of cells that evolved for 550.000 time steps for different values of 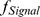 (plot A) and 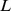 (plot B). The grey area shows the standard deviation. For every parameter setting, 50 independent runs were studied. ‘Signal’ gives the average amount of signal that is present in the environment per time step and colony. 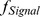 is the reduced chance of having cell division. Thus, 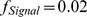 is equal to a 2% lower chance of having cell division. Notice that the maximum chance of having cell division is 10% (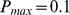). 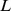 is the number of individuals that initiate a colony and hence the bottleneck size. For plot A we assumed that 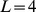 and for plot B we assume that 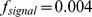. Thus, the runs of plot B at 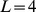 are performed under the same parameter settings as those of plot A at 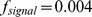. The relatively large standard deviation in plot B can be explained by the co-existence of multiple communicative strategies, of which some produce signal, while others do not. Since the abundances of these strategies change over time, the amount of signal that is being present differs strongly between the runs. Furthermore, in some runs cell-to-cell communication does not evolve (e.g. at high values of 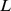). The other parameter settings are the following: 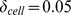, 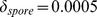, 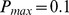, 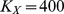, 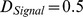, 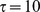, 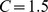, 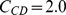, 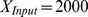, 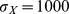, 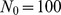, 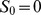, 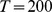, 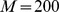, 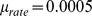 and 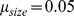.

It is important to notice that the evolutionary simulation shown by figures 5, 6 and 7 assumes relatively low costs for signal production and a small bottleneck size. The costs of signal production are 2% of the maximal growth rate (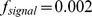), which means that a signal-producing cell has a 2% smaller chance to divide than an undifferentiated cell under the optimal growth conditions. The bottleneck size is given by the number of individuals that initiate a single colony (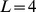). Smaller bottleneck sizes facilitate assortment, because signal-producing cells are more likely to end up in a colony that only contains signal-producers. As a consequence, signal-producing cells are less likely to be exploited by cells that lack signal production. Figure 8 shows how the evolution of cell-to-cell communication depends on 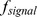 and 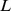, by showing the average amount of signal that is present in a population that evolved for 550.000 time steps. As expected, cell-to-cell communication is more likely to evolve for smaller signal costs and stronger population bottlenecks.

In conclusion, when signal production is costly, cell-to-cell communication can still evolve. However, signal-producing cells can be exploited by cells that lack signal production. This ultimately results in the evolution of ecological diversity, in which multiple ecotypes can coexist. Even though it is to be expected that signal production costs result in cheating (i.e. cells that do not produce signal), it is less intuitive that three ecotypes would evolve, including one that cheats for high nutrient inputs and another that cheats for low nutrient inputs. This coexistence is facilitated by negative frequency-dependent selection, which results from the selection processes at the cellular- and colony-level. Cell-to-cell communication only emerges in our simulations for relatively low costs of signal production and in the presence of population bottlenecks.

## Discussion

We demonstrated that cell-to-cell communication can evolve to regulate the timing of sporulation. The evolution of cell-to-cell communication requires both the evolution of signal production and signal-sensitivity. By sensing quorum-sensing signals a cell can predict future environmental conditions and thereby anticipate a starvation period by initiating sporulation. To predict the environmental conditions a cell has to assess the rate of nutrient consumption, which depends on the colony size. Our model shows that three conditions, which inevitably relate to sporulation, are sufficient to explain the evolution of cell-to-cell communication: (i) the population size has to affect the nutrient concentration (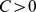); (ii) a cell has to predict future environmental conditions (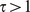; see also [40]–[42]); and (iii) there has to be environmental variation (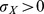). Irrespectively of how these conditions come about, when all three are satisfied and signal production is not too costly, cell-to-cell communication evolves. It is not our claim that these conditions are strictly necessary, but rather that they are sufficient for the evolution of cell-to-cell communication. In nature, the requirements for the evolution of cell-to-cell communication in sporulating bacteria might be less stringent, since additional advantages, besides the timing of cell differentiation, can facilitate the evolution of cell-to-cell communication (e.g. colony-level properties; [2]).

In contrast to previous models on the evolution of cell-to-cell communication [43]–[46], our model shows that cell-to-cell communication can evolve as a mechanism to evaluate other environmental cues [34], [36]: neither the absolute signal concentration nor the absolute nutrient concentration determine the onset of sporulation. To understand when cell-to-cell communication evolves one has to understand how the information that results from quorum-sensing signaling is integrated with that of other environmental cues [47]–[49]. Moreover, we have demonstrated that cell-to-cell communication can even evolve when there is genetic variation within the colony and, in addition, when signal production is costly. Models on sporulation (or other persistence phenotypes) often exclude cell-to-cell communication as a mechanism to regulate sporulation [27], [50], [51]. This is because sporulation is mostly studied as a bet-hedging strategy: only a small fraction of genetically-identical cells sporulates under the same environmental conditions [28], [40]. Bet-hedging is a risk-spreading strategy that ensures the survival of a colony when there are severe and sudden environment changes [52], [53]. In our model a bet-hedging strategy cannot evolve, because cells always perceive accurate environmental information and lack developmental noise. Furthermore, bet-hedging is only beneficial when environmental changes are unpredictable [50], [54]. In our model, environmental changes might only become unpredictable when a cell is surrounded by different ecotypes, which differ in the amount of signal production and the timing of sporulation. It might therefore be interesting to extend the model, in order to examine how the evolution of bet-hedging affects that of cell-to-cell communication.

In our model, cell-to-cell communication represents a form of phenotypic plasticity, because it allows a cell to adjust the timing of sporulation in response to environmental changes [55]. Without cell-to-cell communication a cell can only grow efficiently for a limited range of nutrient inputs (figure S5). In that case, multiple ecotypes evolve that specialize on distinct ecological niches (e.g. the late and early sporulating ecotypes that evolved at the onset of our simulations, see figure 3C–D). However, by evolving cell-to-cell communication the range of nutrient inputs at which a cell grows efficiently increases. This ultimately results in competitive exclusion: the specialized ecotypes (i.e. narrow niche width)—such as the late and early sporulating ecotypes—are replaced by a single generalist (i.e. broad niche width) that can grow efficiently under most environment conditions due to cell-to-cell communication [56]–[58]. In our model phenotypic plasticity is a colony-level property, instead of a cellular property, since cells cannot respond to changes in environmental conditions without cooperation [59]: the amount of signal only gives an accurate indication of the colony size when all cells (or a constant fraction) produce quorum-sensing signals. The evolution of cell-to-cell communication therefore entails a cooperative dilemma (given that signal production is costly; [4], [60]–[62]). Cells that do not produce signal (i.e. public good) have an advantage over those that do, but at the same time they undermine the colony performance (see also [4], [63]–[66]). The cells that do not produce signal could therefore be called ‘cheaters’, while signal-producing cells are ‘cooperators’.

In our model, cheaters and cooperators evolved and stably coexisted due to frequency-dependent selection [43], [46], [67]–[71]. They have different communicative strategies [72] and therefore occupy distinct complementary niches (see figure 5 and 6). That is, the cheaters lack signal production for different subsets of environmental conditions. This emphasizes the importance of studying cell-to-cell communication under a wide range of environmental conditions, since a cooperator under one condition might be a cheater under another. The population structure (see figure 1), which results in two levels of selection, was essential for the maintenance of the different ecotypes [66], [73], [74]. Previous studies have shown that population structure can facilitate the evolution and maintenance of cooperation [69], [75]–[83]. The population structure makes individuals interact assortatively [84]: cooperators are therefore more likely to interact with other cooperators than cheaters. As a consequence, the benefits of cooperation mostly end up with cooperators, due to which there is a net selective advantage for cooperation. In our model the degree of assortment depends on the number of individuals that initialize a single colony (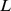) or, in other words, on the strength of the recurrent population bottlenecks [85], . We assumed that the colonies themselves are well-mixed, although within-colony structure—via the emergence of assortment—might have facilitated cooperation even more [87], [88]. When signal production is too costly, cell-to-cell communication does not evolve, because the selective advantage of cheaters at the cellular-level cannot be compensated by the selective advantage of cooperators at the colony-level. It is important to notice that our model only included signal production costs, even though plausible arguments could be made that the maintenance costs of a communicative system should be considered as well [89]. However, we do not expect that including maintenance costs would affect our results, since both cheaters and cooperators need to have a communicative system—and hence carry the associated costs—to sense the quorum-sensing signal.

Although our model is limited to sporulation, it could be extended to examine the role of cell-to-cell communication in the timing of other differentiation events as well, for example: motility, bioluminescence, conjugation, competence, matrix-production, biofilm formation, biofilm detachment, etc. (e.g. [11]–[14], [47], [90]–[94]). Every time there is a trade-off between the growth rate of two cell types (e.g. cells and spores) over two or more environmental niches that alternate over time (e.g. nutrient availability and nutrient scarcity), a cell has a selective advantage when it accurately times the developmental transitions between both cell types (see also [45]). When the population size affects the optimal time at which a cell should differentiate (e.g. when a cell must predict future nutrient conditions), cell-to-cell communication is expected to evolve in order to enhance a cell's developmental timing. The challenge for future studies is to unravel the developmental trade-off and ecological niches that underlie each of these differentiation events. Furthermore, our study emphasizes the importance of examining the integration of different environmental cues in cellular decision-making [49], [95]–[97]. The quorum-sensing threshold—and hence the critical population density—at which a differentiation event occurs can and mostly will strongly depend on other environmental conditions, such as nutrient availability [34], [48], [98], [99].

## Supporting Information

Figure S1
**The evolution of cell-to-cell communication under clonal growth conditions.** These plots depict the evolutionary trajectories of 100 runs performed under the same conditions than those shown in figure 3A. The left plot shows for every 20.000 time steps the average evolved genotype, which is given by the mean value of 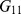 and 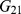 over 100 runs. The error bars show the standard deviations. In total 600.000 time steps of evolution are shown; starting from the *dark-blue* dot till the *red* dot. The right plot shows a subset of runs that evolved cell-to-cell communication, using the same color coding. For parameter settings see figure 3 of the main text.(TIF)

Figure S2
**The evolution of cell-to-cell communication under non-clonal growth conditions.** These plots show the evolved values of 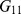 for 18 independent evolutionary runs. The conditions of these runs are the same as those shown in figure 3D, in which a early and late sporulating ecotype evolved. The simulations ran for 600.000 time steps, of which 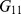 is shown for the most-abundant genotypes (present in the population in more than 100 copies) at 5.000 time steps intervals. For parameter settings see figure 3 of the main text.(TIF)

Figure S3
**The evolution of cell-to-cell communication when the activation thresholds can evolve.** The plots show the evolution of cell-to-cell communication under the same conditions as those shown in figure S1, S2 and 3. However, in contrast to these previous figures, the simulations in this figure allowed for the evolution of the activation thresholds (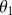 and 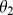). To facilitate a comparison between the plots in this figure and figure S1, S2 and 3, all shown connection weights are corrected for the evolved activation threshold. This is done by dividing the connection weights by two times the absolute value of the associated activation threshold (notice that for the previous figures we assumed that 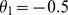). Plot A and B show the evolution of cell-to-cell communication under clonal growth conditions (see figure S1). Plot A shows for every 20.000 time steps the average evolved genotype, which is given by the mean value of 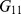 and 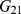 over 100 runs. The error bars show the standard deviations. In total 600.000 time steps of evolution are shown; starting from the *dark-blue* dot till the *red* dot. Plot B shows a subset of runs that evolved cell-to-cell communication, using the same color coding. Plot C shows the evolution of cell-to-cell communication under non-clonal growth conditions. The subplots show the evolved values of 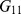 for the most-abundant genotypes (present in the population in more than 100 copies) at 5.000 time steps intervals. Only 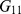 is shown, since this illustrates the evolution of the early and late sporulating ecotype, as shown in figure 3 and S2. For parameter settings see figure 3 of the main text.(TIF)

Figure S4
**Growth of mixed colonies, consisting of early and late sporulating genotypes, at high and low nutrient inputs.** The left plots show colony growth at a low nutrient input (i.e. 1000) and the right plots show the same for a high nutrient input (i.e. 2500). The early and late sporulating genotypes shown here do not have cell-to-cell communication and only differ with respect to the nutrient concentration at which sporulation is initiated: the early sporulating genotype sporulates at a nutrient concentration of 1000 (*blue* dashed line in the upper plot), while the late sporulating genotype sporulates at a nutrient concentration of 500 (*red* dashed line in the upper plot). The upper plots show the nutrient concentration (*green* line), the middle plots show the number of cells and the lower plots show the number of spores of the early (*blue*) and late (*red*) sporulating genotypes. Each line is the average of 1000 replicate runs and the shaded area shows the associated standard deviation. The parameter settings that are used for these simulations are the following: 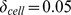, 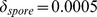, 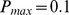, 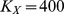, 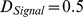, 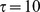, 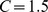, 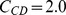, 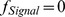, 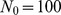, 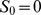 and 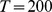.(TIF)

Figure S5
**Overview of the colony performance at different nutrient inputs (i.e. nutrient concentration at onset of colony growth).** Plot A: the distribution of nutrient inputs from which the nutrient input of a colony is taken in the evolutionary simulations (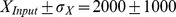). Plot B: The average colony size at the end of colony growth for two different genotypes that do not use cell-to-cell communication. The *red* line shows the average colony size for a late sporulating genotype (this genotype initiates sporulation when 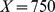) and the *blue* line shows the average colony size for an early sporulating genotype (this genotype initiates sporulation when 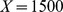). The dotted lines show the standard deviation from the average at each nutrient input. Plot C: The average colony size at the end of colony growth for the evolved genotype of figure 3. The evolved genotype is the dominant genotype that is present at the end of the simulation. This genotype evolved cell-to-cell communication (figure 3D) and has the following genotype: 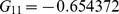; 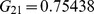; 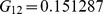; 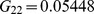; 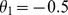 and 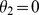. Even though the optimal genotype is not reached yet (see figure 3C), this genotype performs considerably well under all possible nutrient inputs.(TIF)

Figure S6
**Invasion analysis of early and late sporulating genotype.** The relative fitness of the early and late sporulating genotypes for different starting conditions: (A) 10% of early sporulating genotypes or (B) 10% of late sporulating genotypes. When a genotype's fitness is higher than one it is favored by selection and when it is lower than one it is selected against. There is frequency-dependent selection, since each genotype has a fitness advantage when it is rare: the early sporulating genotype has a fitness advantage when it is rare (A) and the late sporulating genotype has a fitness advantage when it is rare (B). The genotypes only differ in their sensitivity towards the nutrient concentration: 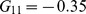 for the early sporulating genotype and 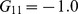 for the late sporulating genotype. Each bar shows the average fitness over 10 replicates and the error bars show the standard deviation. Each replicate consists of 200 colonies which are grown under the following conditions: 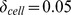, 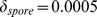, 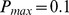, 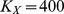, 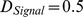, 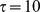, 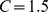, 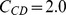, 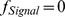, 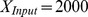, 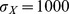, 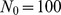, 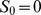, 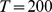 and 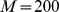.(TIF)

Figure S7
**Pairwise invasibility plots for different levels of environmental variation.** Each plot shows the invasibility of a mutant, given the presence a certain resident population. The genotypes differ in the nutrient concentration at which sporulation is initiated, which is shown for the resident genotype on the x-axis and for the mutant genotype on the y-axis. For each combination of mutant and resident, the invasibility of the mutant is tested by growing 800 colonies that are initiated with 10% of mutants. The mutant is said to invade when its average fitness is higher then that of the resident (*red* area), while it goes extinct when its fitness is lower (*blue* area). The black diagonal line shows when the resident and mutant have the same genotype and hence fitness. The different plots show the invasibility for various levels of environmental variation: 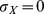, 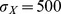 or 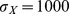. For 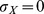 there is an evolutionary stable strategy (ESS) that cannot be invaded by mutants. This is illustrated by the *white* dot on the black diagonal line (the ESS is a resident genotype that sporulates at a nutrient concentration around 1100). The vertical *white* line, which is associated with the ESS, occurs exclusively in the *blue* region of the plot. This shows that none of the mutants can invade the ESS resident population. For 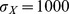 there is no ESS (as illustrated by the *black* dot), since one cannot draw a vertical *white* line that exclusively occurs in the *blue* region of the plot. 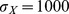 is also the condition for which we observe branching in the evolutionary simulations (see figure 3D and S2). The parameter settings that are used for these simulations are the following: 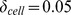, 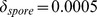, 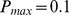, 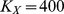, 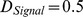, 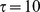, 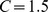, 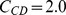, 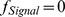, 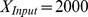, 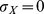, 500 or 1000, 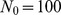, 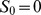, 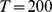 and 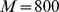.(TIF)

Figure S8
**Selective advantage of cell-to-cell communication when varying the initial colony size.** The relative fitness benefit of quorum-sensing signaling under different levels of variation in the initial colony size. In all other simulations we assumed that the initial colony size is constant. However, here we vary the initial colony size, which is taken from a normal distribution 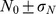. The normal distribution of the colony size is truncated at 10 individuals, so that no colony was initiated with less than 10 individuals. The standard deviation is shown on the x-axis of this plot. Furthermore, we assume that there is no variation in the initial nutrient concentration (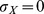). The bars show the average fitness of a quorum-sensing cell, relative to that of a cell that does not communicate. The error bars show the standard deviation over 100 replicate runs, each containing 200 colonies. When the relative fitness of a quorum-sensing cell is equal to 1 (the horizontal *black* line), there is no selective advantage for cell-to-cell communication. When it is higher than 1 there is a selective advantage for cell-to-cell communication. The parameter settings are the following: 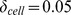, 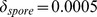, 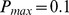, 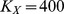, 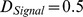, 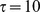, 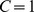, 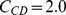, 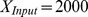, 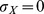, 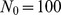, 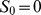 and 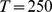.(TIF)

Figure S9 **Unrooted phenograms.** Four replicate studies for the evolution of cell-to-cell communication under the assumption of costly signal production. The simulations are performed under the same conditions as those shown in figure 5 of the main text and also the phenograms are constructed in accordance to figure 5. As shown in the main text, different genotypic clusters evolved and coexist, which consist of ‘cooperative’ and ‘cheating’ phenotypes. For each genotypic cluster a single representative phenotype is shown, which is produced by the most-abundant genotype within this cluster. For details see figure 5 of the main text.
(TIF)

Table S1
**The number of cells that are present at the end of colony growth for each phenotype, given the initial colony composition.** On the left side of the table the colony composition is shown, because 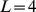 each phenotype could occur in 0%, 25%, 50%, 75% or 100% of the initial colony composition. The right side of the table shows the average number of cells that are present at the end of colony growth. The standard deviation is taken over 4 replicates, each replicate contains 200 colonies that are initiated with a nutrient input that is taken from the normal distribution, 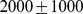 (for the 4 replicates the same nutrient inputs are used).(PDF)

Text S1 **Supplementary material.**
(PDF)
