# Supplementary figures and images for: The Evolution of Cell-to-Cell Communication in a Sporulating Bacterium
Source: PLoS Comput Biol. 2012 Dec 20;8(12):e1002818. doi: 10.1371/journal.pcbi.1002818 (PMC3527279; doi:10.1371/journal.pcbi.1002818)

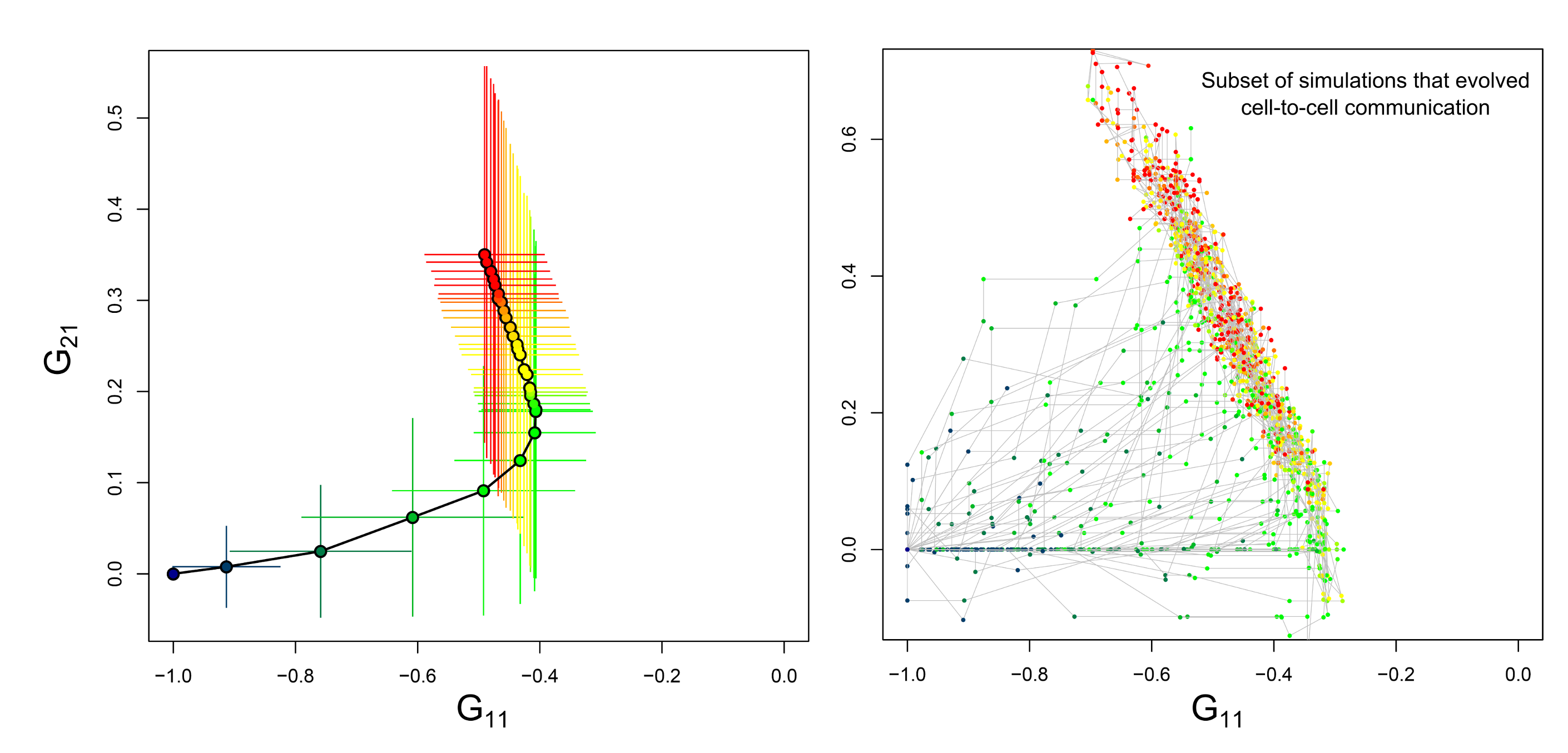

Supplement: Figure S1 — The evolution of cell-to-cell communication under clonal growth conditions. These plots depict the evolutionary trajectories of 100 runs performed under the same conditions than those shown in figure 3A. The left plot shows for every 20.000 time steps the average evolved genotype, which is given by the mean value of and over 100 runs. The error bars show the standard deviations. In total 600.000 time steps of evolution are shown; starting from the dark-blue dot till the red dot. The right plot shows a subset of runs that evolved cell-to-cell communication, using the same color coding. For parameter settings see figure 3 of the main text. (TIF) [file pcbi.1002818.s001.tif]

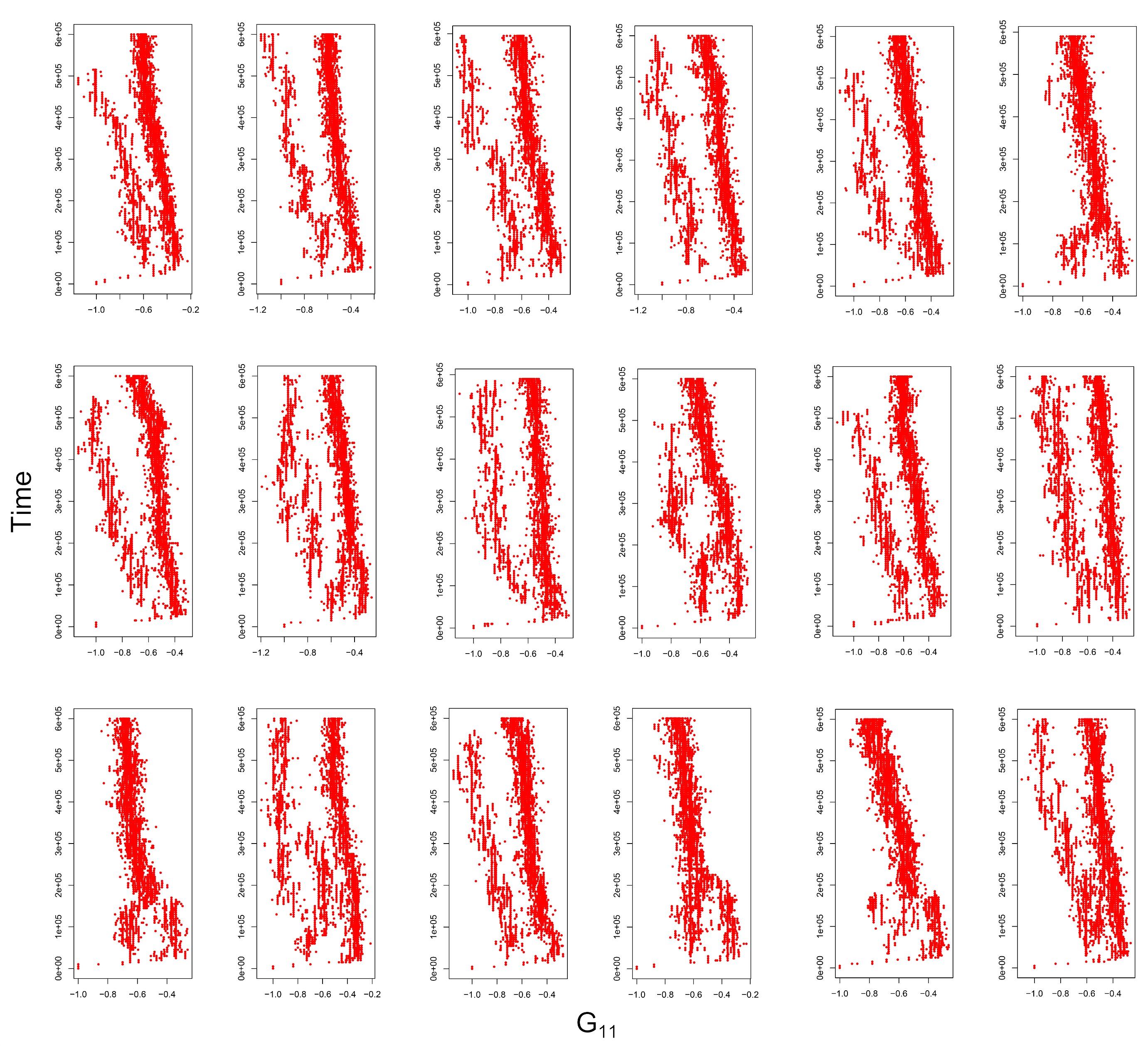

Supplement: Figure S2 — The evolution of cell-to-cell communication under non-clonal growth conditions. These plots show the evolved values of for 18 independent evolutionary runs. The conditions of these runs are the same as those shown in figure 3D, in which a early and late sporulating ecotype evolved. The simulations ran for 600.000 time steps, of which is shown for the most-abundant genotypes (present in the population in more than 100 copies) at 5.000 time steps intervals. For parameter settings see figure 3 of the main text. (TIF) [file pcbi.1002818.s002.tif]

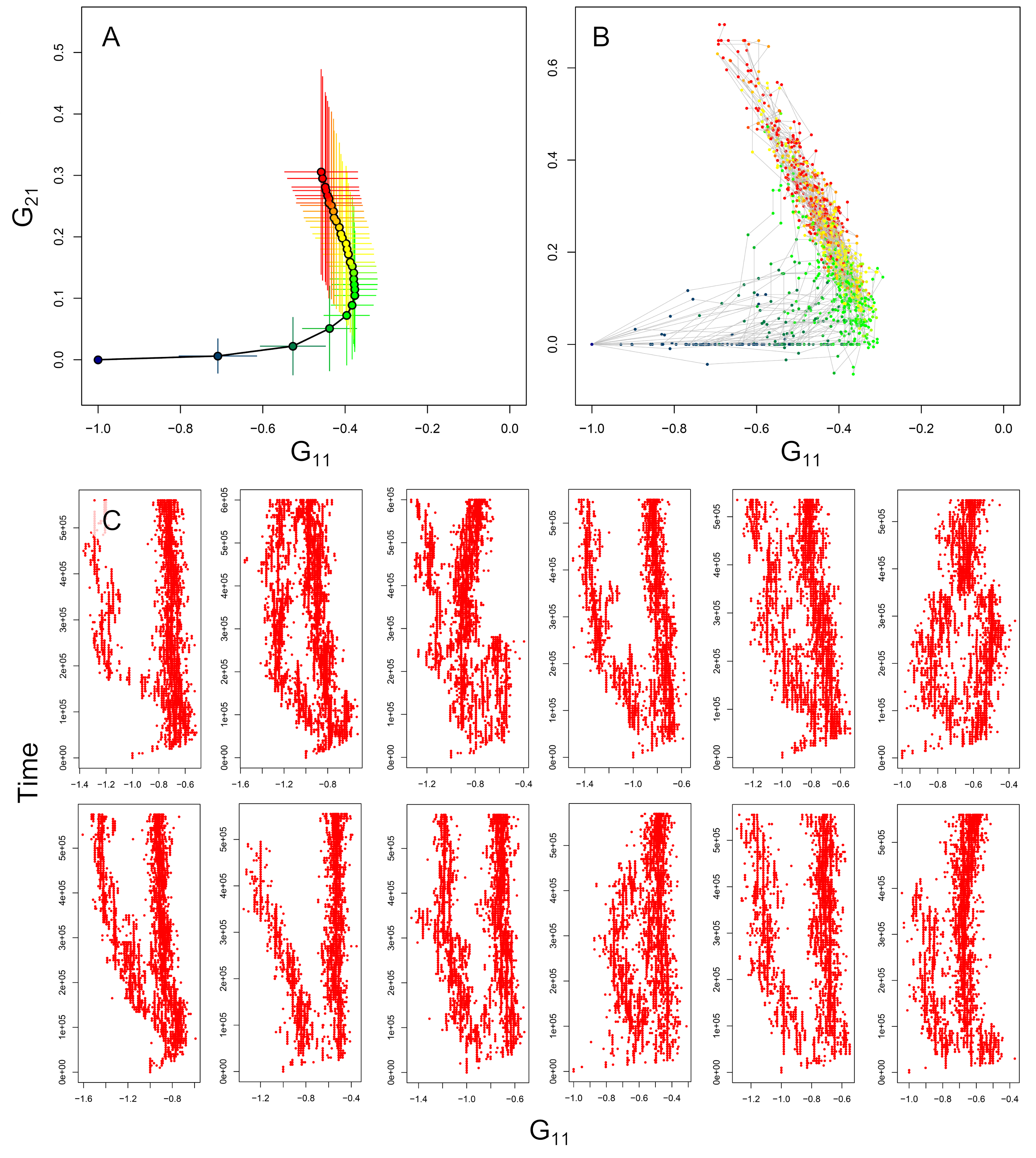

Supplement: Figure S3 — The evolution of cell-to-cell communication when the activation thresholds can evolve. The plots show the evolution of cell-to-cell communication under the same conditions as those shown in figure S1, S2 and 3. However, in contrast to these previous figures, the simulations in this figure allowed for the evolution of the activation thresholds ( and ). To facilitate a comparison between the plots in this figure and figure S1, S2 and 3, all shown connection weights are corrected for the evolved activation threshold. This is done by dividing the connection weights by two times the absolute value of the associated activation threshold (notice that for the previous figures we assumed that ). Plot A and B show the evolution of cell-to-cell communication under clonal growth conditions (see figure S1). Plot A shows for every 20.000 time steps the average evolved genotype, which is given by the mean value of and over 100 runs. The error bars show the standard deviations. In total 600.000 time steps of evolution are shown; starting from the dark-blue dot till the red dot. Plot B shows a subset of runs that evolved cell-to-cell communication, using the same color coding. Plot C shows the evolution of cell-to-cell communication under non-clonal growth conditions. The subplots show the evolved values of for the most-abundant genotypes (present in the population in more than 100 copies) at 5.000 time steps intervals. Only is shown, since this illustrates the evolution of the early and late sporulating ecotype, as shown in figure 3 and S2. For parameter settings see figure 3 of the main text. (TIF) [file pcbi.1002818.s003.tif]

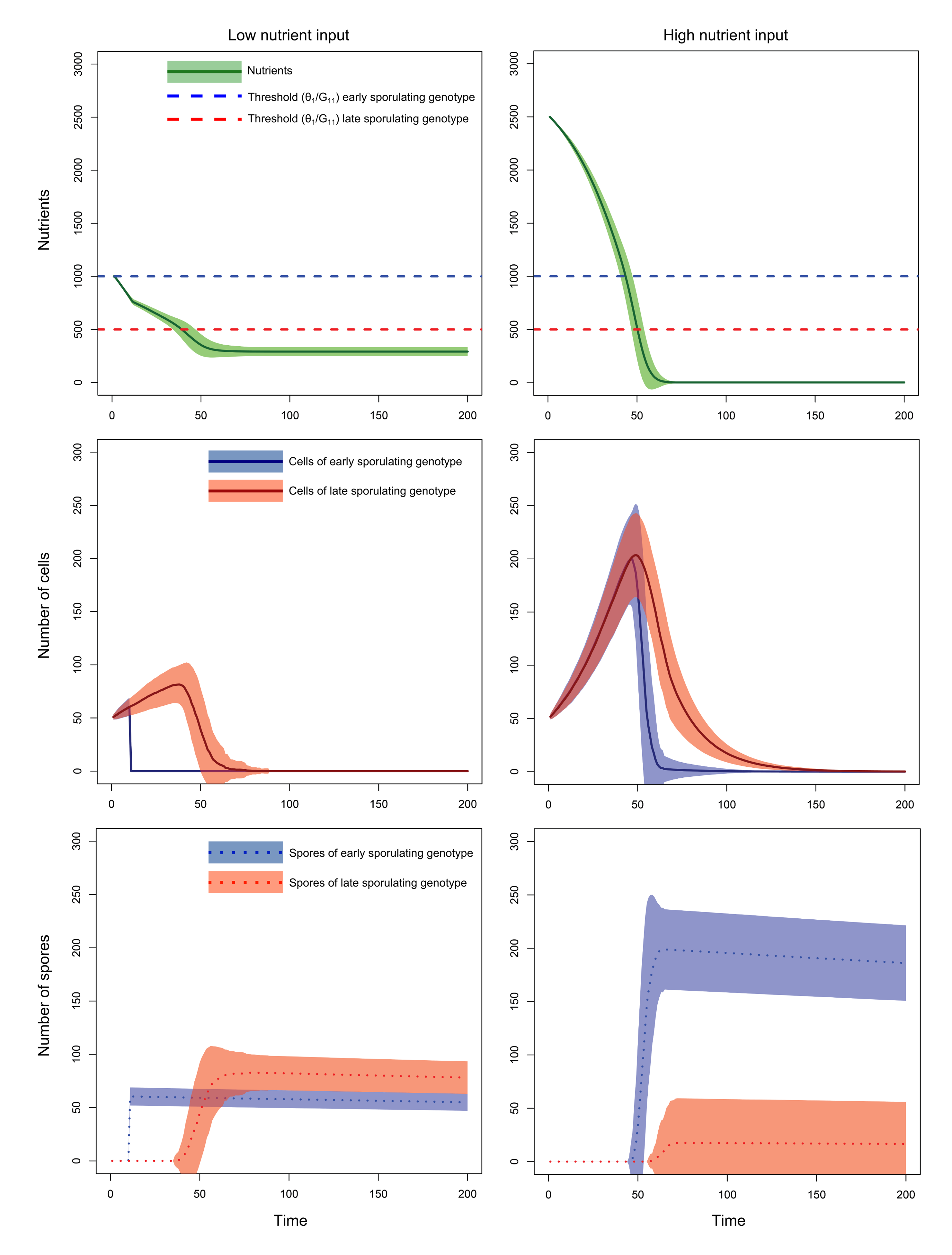

Supplement: Figure S4 — Growth of mixed colonies, consisting of early and late sporulating genotypes, at high and low nutrient inputs. The left plots show colony growth at a low nutrient input (i.e. 1000) and the right plots show the same for a high nutrient input (i.e. 2500). The early and late sporulating genotypes shown here do not have cell-to-cell communication and only differ with respect to the nutrient concentration at which sporulation is initiated: the early sporulating genotype sporulates at a nutrient concentration of 1000 (blue dashed line in the upper plot), while the late sporulating genotype sporulates at a nutrient concentration of 500 (red dashed line in the upper plot). The upper plots show the nutrient concentration (green line), the middle plots show the number of cells and the lower plots show the number of spores of the early (blue) and late (red) sporulating genotypes. Each line is the average of 1000 replicate runs and the shaded area shows the associated standard deviation. The parameter settings that are used for these simulations are the following: , , , , , , , , , , and . (TIF) [file pcbi.1002818.s004.tif]

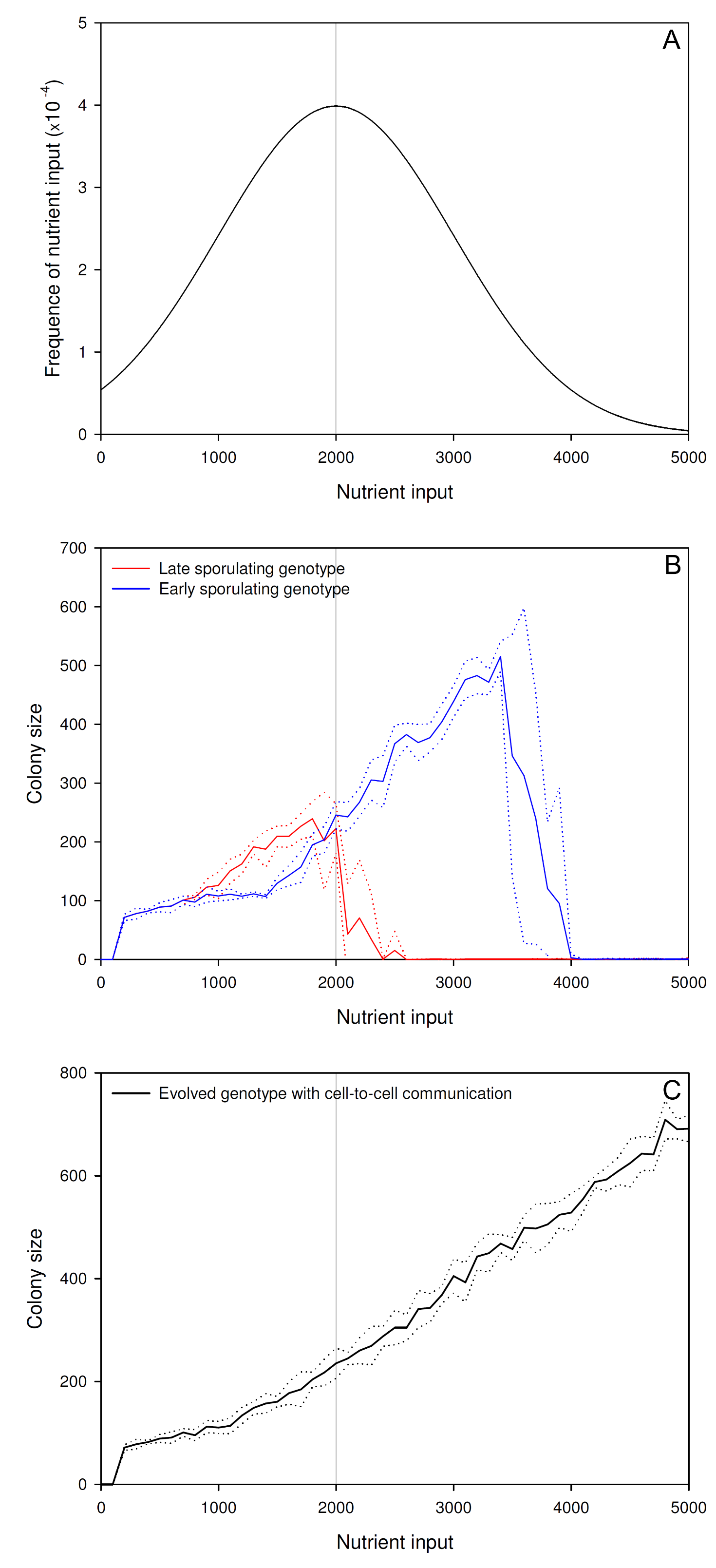

Supplement: Figure S5 — Overview of the colony performance at different nutrient inputs (i.e. nutrient concentration at onset of colony growth). Plot A: the distribution of nutrient inputs from which the nutrient input of a colony is taken in the evolutionary simulations (). Plot B: The average colony size at the end of colony growth for two different genotypes that do not use cell-to-cell communication. The red line shows the average colony size for a late sporulating genotype (this genotype initiates sporulation when ) and the blue line shows the average colony size for an early sporulating genotype (this genotype initiates sporulation when ). The dotted lines show the standard deviation from the average at each nutrient input. Plot C: The average colony size at the end of colony growth for the evolved genotype of figure 3. The evolved genotype is the dominant genotype that is present at the end of the simulation. This genotype evolved cell-to-cell communication (figure 3D) and has the following genotype: ; ; ; ; and . Even though the optimal genotype is not reached yet (see figure 3C), this genotype performs considerably well under all possible nutrient inputs. (TIF) [file pcbi.1002818.s005.tif]

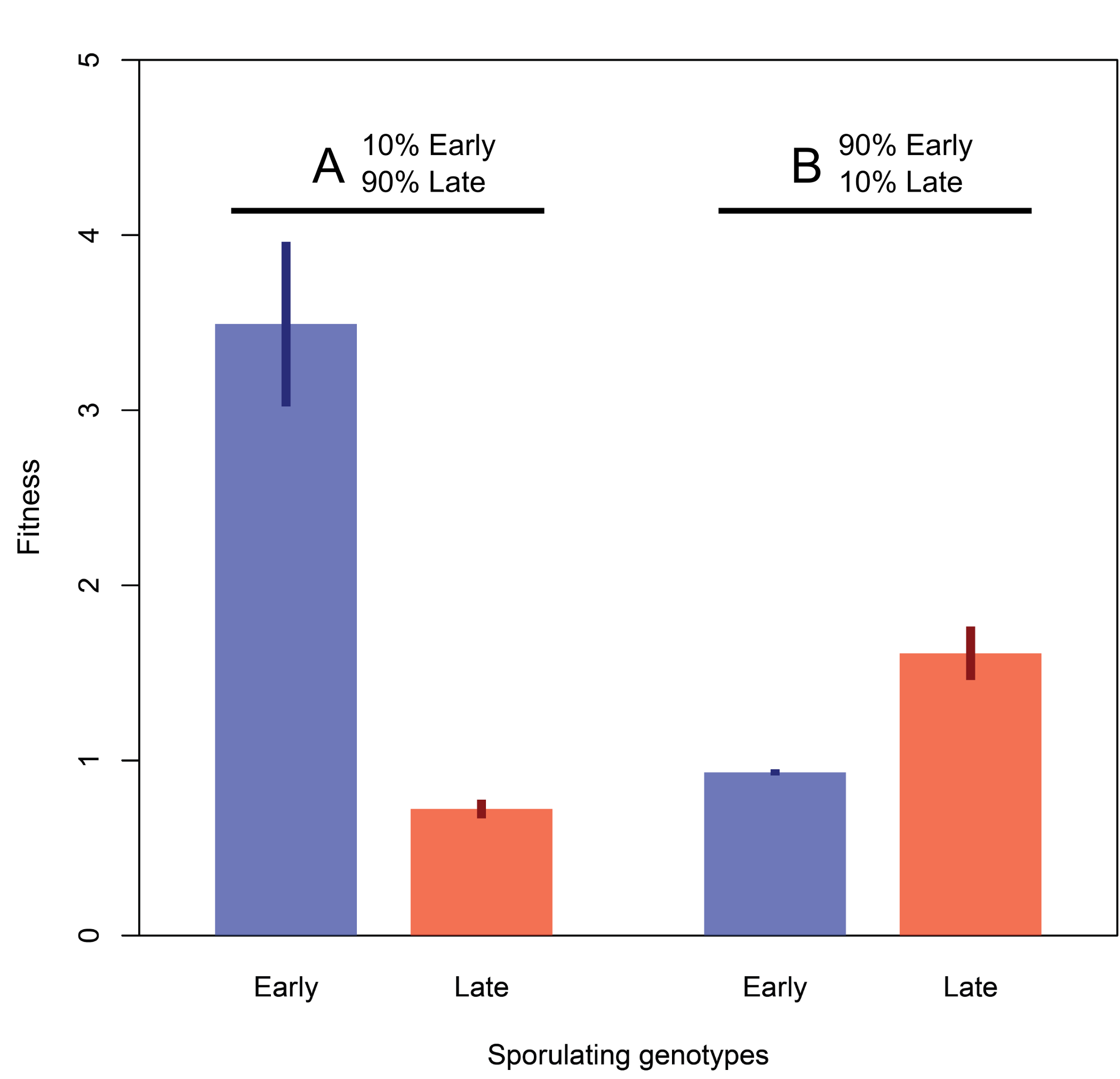

Supplement: Figure S6 — Invasion analysis of early and late sporulating genotype. The relative fitness of the early and late sporulating genotypes for different starting conditions: (A) 10% of early sporulating genotypes or (B) 10% of late sporulating genotypes. When a genotype's fitness is higher than one it is favored by selection and when it is lower than one it is selected against. There is frequency-dependent selection, since each genotype has a fitness advantage when it is rare: the early sporulating genotype has a fitness advantage when it is rare (A) and the late sporulating genotype has a fitness advantage when it is rare (B). The genotypes only differ in their sensitivity towards the nutrient concentration: for the early sporulating genotype and for the late sporulating genotype. Each bar shows the average fitness over 10 replicates and the error bars show the standard deviation. Each replicate consists of 200 colonies which are grown under the following conditions: , , , , , , , , , , , , , and . (TIF) [file pcbi.1002818.s006.tif]

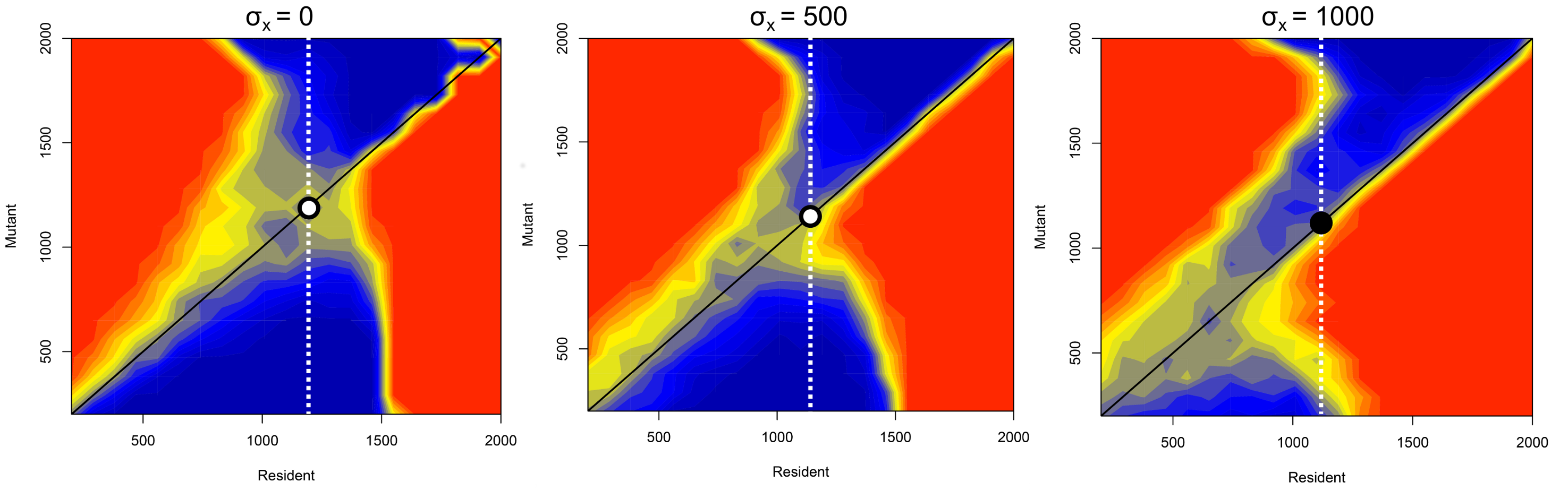

Supplement: Figure S7 — Pairwise invasibility plots for different levels of environmental variation. Each plot shows the invasibility of a mutant, given the presence a certain resident population. The genotypes differ in the nutrient concentration at which sporulation is initiated, which is shown for the resident genotype on the x-axis and for the mutant genotype on the y-axis. For each combination of mutant and resident, the invasibility of the mutant is tested by growing 800 colonies that are initiated with 10% of mutants. The mutant is said to invade when its average fitness is higher then that of the resident (red area), while it goes extinct when its fitness is lower (blue area). The black diagonal line shows when the resident and mutant have the same genotype and hence fitness. The different plots show the invasibility for various levels of environmental variation: , or . For there is an evolutionary stable strategy (ESS) that cannot be invaded by mutants. This is illustrated by the white dot on the black diagonal line (the ESS is a resident genotype that sporulates at a nutrient concentration around 1100). The vertical white line, which is associated with the ESS, occurs exclusively in the blue region of the plot. This shows that none of the mutants can invade the ESS resident population. For there is no ESS (as illustrated by the black dot), since one cannot draw a vertical white line that exclusively occurs in the blue region of the plot. is also the condition for which we observe branching in the evolutionary simulations (see figure 3D and S2). The parameter settings that are used for these simulations are the following: , , , , , , , , , , , 500 or 1000, , , and . (TIF) [file pcbi.1002818.s007.tif]

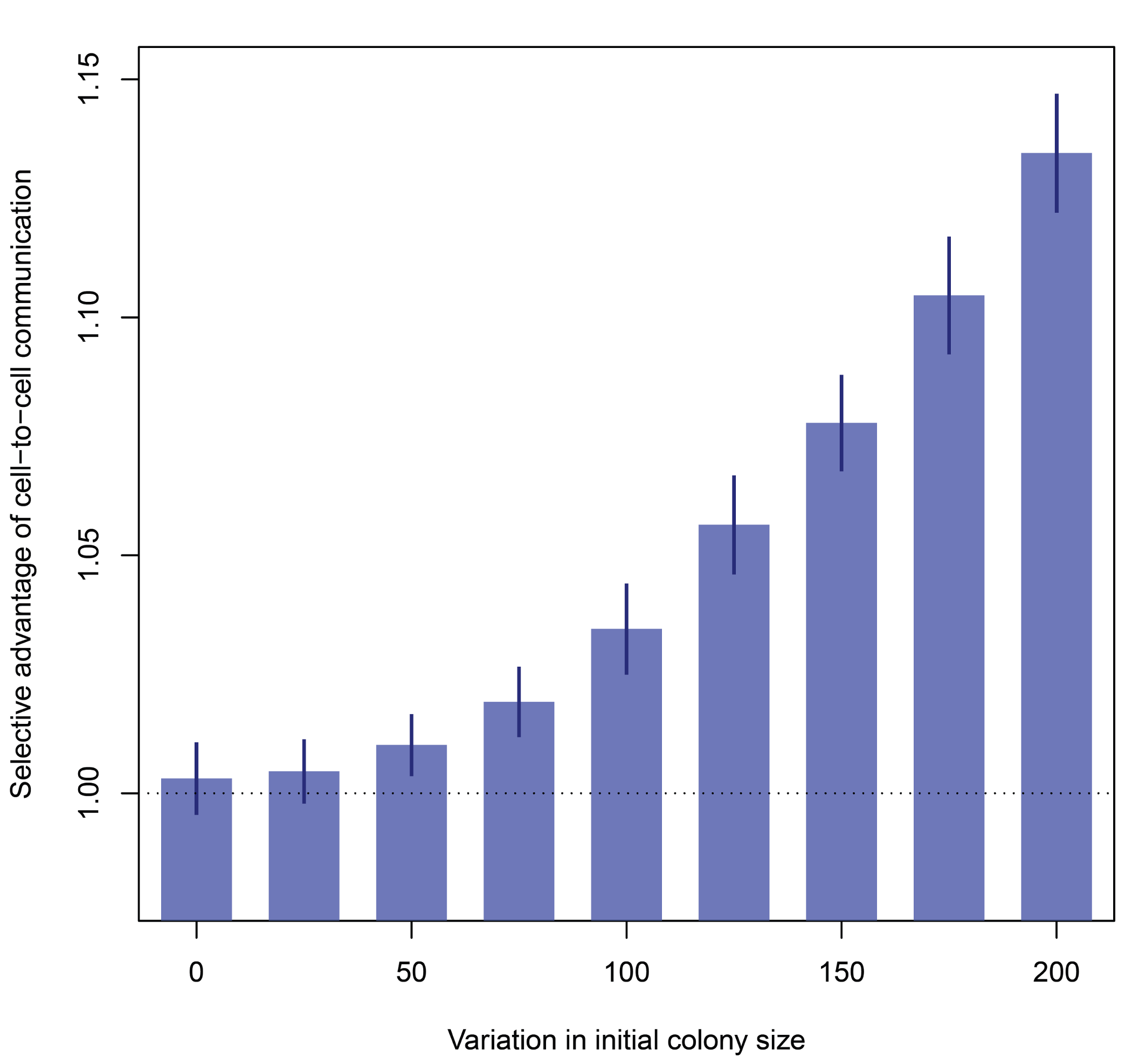

Supplement: Figure S8 — Selective advantage of cell-to-cell communication when varying the initial colony size. The relative fitness benefit of quorum-sensing signaling under different levels of variation in the initial colony size. In all other simulations we assumed that the initial colony size is constant. However, here we vary the initial colony size, which is taken from a normal distribution . The normal distribution of the colony size is truncated at 10 individuals, so that no colony was initiated with less than 10 individuals. The standard deviation is shown on the x-axis of this plot. Furthermore, we assume that there is no variation in the initial nutrient concentration (). The bars show the average fitness of a quorum-sensing cell, relative to that of a cell that does not communicate. The error bars show the standard deviation over 100 replicate runs, each containing 200 colonies. When the relative fitness of a quorum-sensing cell is equal to 1 (the horizontal black line), there is no selective advantage for cell-to-cell communication. When it is higher than 1 there is a selective advantage for cell-to-cell communication. The parameter settings are the following: , , , , , , , , , , , and . (TIF) [file pcbi.1002818.s008.tif]

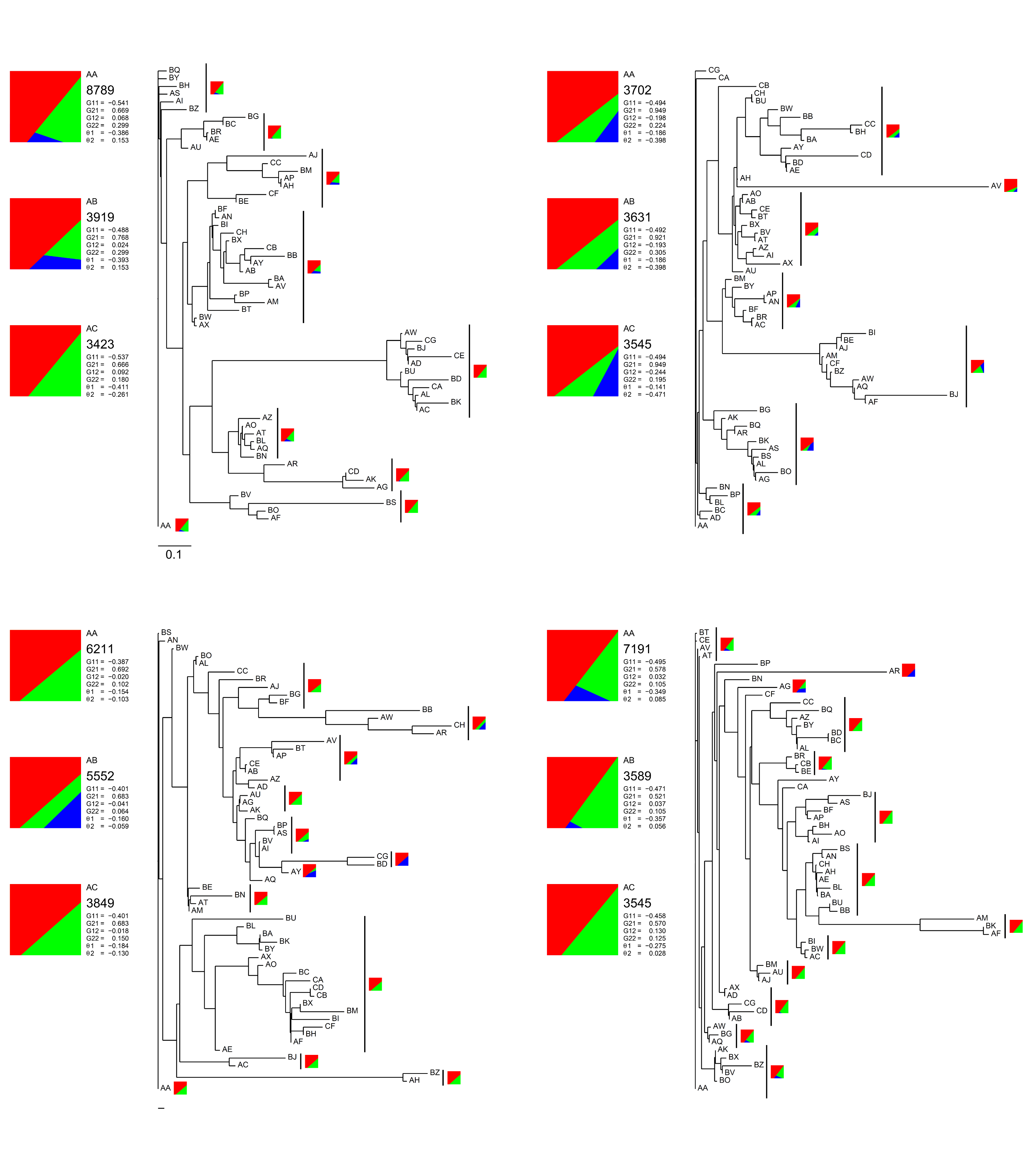

Supplement: Figure S9 — Unrooted phenograms. Four replicate studies for the evolution of cell-to-cell communication under the assumption of costly signal production. The simulations are performed under the same conditions as those shown in figure 5 of the main text and also the phenograms are constructed in accordance to figure 5. As shown in the main text, different genotypic clusters evolved and coexist, which consist of ‘cooperative’ and ‘cheating’ phenotypes. For each genotypic cluster a single representative phenotype is shown, which is produced by the most-abundant genotype within this cluster. For details see figure 5 of the main text. (TIF) [file pcbi.1002818.s009.tif]
