# Supplementary material for: The Evolution of Cell-to-Cell Communication in a Sporulating Bacterium
Source: PLoS Comput Biol. 2012 Dec 20;8(12):e1002818. doi: 10.1371/journal.pcbi.1002818 (PMC3527279; doi:10.1371/journal.pcbi.1002818)
Supplement: Table S1 — The number of cells that are present at the end of colony growth for each phenotype, given the initial colony composition. On the left side of the table the colony composition is shown, because each phenotype could occur in 0%, 25%, 50%, 75% or 100% of the initial colony composition. The right side of the table shows the average number of cells that are present at the end of colony growth. The standard deviation is taken over 4 replicates, each replicate contains 200 colonies that are initiated with a nutrient input that is taken from the normal distribution, (for the 4 replicates the same nutrient inputs are used). (PDF) [file pcbi.1002818.s010.pdf]

Table S1: **The number of cells that are present at the end of colony growth for each phenotype, given the initial colony composition.**

Initial frequency (%)			Number of cells at the end of colony growth			
Pheno 1	Pheno 2	Pheno 3	Phenotype 1	Phenotype 2	Phenotype 3	Total
0	0	100	0 ± 0	0 ± 0	197.2 ± 3.2	197.2 ± 3.2
0	25	75	0 ± 0	66.2 ± 0.7	207.4 ± 4.6	273.6 ± 4.2
0	50	50	0 ± 0	139.6 ± 3.5	143.1 ± 2.3	282.7 ± 2.9
0	75	25	0 ± 0	208.8 ± 1.7	71.3 ± 1.4	280.2 ± 2.0
0	100	0	0 ± 0	273.6 ± 0.8	0 ± 0	273.6 ± 0.8
25	0	75	65.6 ± 2.0	0 ± 0	201.9 ± 0.8	267.6 ± 1.6
25	25	50	72.5 ± 0.9	69.5 ± 0.7	143.3 ± 3.3	285.3 ± 1.9
25	50	25	71.3 ± 0.9	139.0 ± 2.8	70.7 ± 1.5	281.1 ± 1.2
25	75	0	72.0 ± 1.9	205.7 ± 2.5	0 ± 0	277.7 ± 0.7
50	0	50	142.8 ± 2.2	0 ± 0	143.3 ± 2.4	286.1 ± 1.2
50	25	25	143.9 ± 3.1	69.0 ± 2.3	70.3 ± 1.0	283.3 ± 2.5
50	50	0	143.4 ± 1.7	134.2 ± 2.9	0 ± 0	277.6 ± 1.9
75	0	25	203.2 ± 2.7	0 ± 0	65.2 ± 1.8	268.4 ± 1.4
75	25	0	207.8 ± 2.7	68.1 ± 1.3	0 ± 0	275.9 ± 2.7
100	0	0	251.2 ± 1.5	0 ± 0	0 ± 0	251.2 ± 1.5
