# Supplementary material for: The Evolution of Cell-to-Cell Communication in a Sporulating Bacterium
Source: PLoS Comput Biol. 2012 Dec 20;8(12):e1002818. doi: 10.1371/journal.pcbi.1002818 (PMC3527279; doi:10.1371/journal.pcbi.1002818)
Supplement: Text S1 — Supplementary material. (PDF) [file pcbi.1002818.s011.pdf]

Text S1

The chance of having cell division is the same for all cell types, excluding spores (the cell state of sporulation at which $t = \tau$). Spores cannot divide. The chance of having cell division (P_{CD}) is given by the following equation (in accordance to Monod saturation kinetics):

$$P_{CD} = \frac{P_{max} \cdot X}{X + K_X} \tag{S1}$$

X is the nutrient concentration that is present in the environment, which changes over time. P_{max} is the maximal chance of having cell division, which is the chance of having cell division when there is a saturation in the nutrient concentration ($X \to \infty$). K_X determines how fast—meaning for which increase in the nutrient concentration—the chance of having cell division is saturated. When $X = K_X$, the chance of having cell division equals $\frac{1}{2} \cdot P_{max}$. In contrast to the chance of having cell division, the chance of having cell death is independent of the nutrient concentration and fixed for both cells (δ_{Cell}) and spores (δ_{Spore}). A cell should turn into a spore when the growth rate of a cell exceeds that of a spore. The effective growth rate is the chance of having cell division minus the chance of having cell death. Therefore one could derive the critical nutrient concentration (X^*) at which a cell should turn into a spore:

$$X^* = \frac{(\delta_{cell} - \delta_{spore}) \cdot K_X}{P_{max} - (\delta_{cell} - \delta_{spore})} \tag{S2}$$

When signal production is assumed to be costly, the chance that a signal-producing cell divides is given by:

$$P_{CD,\ Signal\ production} = \frac{P_{max} \cdot X}{X + K_X} - f_{Signal} \tag{S3}$$
